# Switchable quantum dot photoluminescence across a broad spectrum with all-visible-light activated diarylethene photoswitches

**DOI:** 10.1038/s41467-026-78224-2

**Published:** 2026-10-02

**Authors:** Lin Xi, Kezhou Chen, Shuai Zhang, Taibin Wang, Tiegen Liu, Bo Albinsson, Lili Hou

**Affiliations:** 1 State Key Laboratory of Precision Measurement Technology and Instruments, Key Laboratory of Opto-electronics Information Technology, School of Precision Instrument and Opto-Electronics Engineering, Tianjin University, Tianjin, China; 2 Department of Chemistry and Chemical Engineering, Chalmers University of Technology, Gothenburg, Sweden

**Keywords:** Photochemistry, Fluorescence spectroscopy, Quantum dots

## Abstract

Precise and reversible photoluminescence switching is highly attractive for optical memory, bioimaging, and smart displays. However, achieving broadband photoluminescence modulation through a simple and general strategy remains challenging, while the ultraviolet light requirement of most molecular photoswitches limits practical applications. Here, we achieve all-visible-light activated reversible photoluminescence modulation of quantum dots across an ultrawide spectral range of nearly 500 nm from blue to near-infrared region using diarylethene photoswitches. In the ring-open form of diarylethenes, quantum dots emitting blue, green, red, white and near-infrared light remain in the photoluminescence on-state. Under 405 nm irradiation, diarylethenes undergo ring-closure through triplet sensitization by CdS quantum dots, resulting in significant photoluminescence quenching to the off-state, with the maximum efficiency of 99.6% and an on/off ratio up to 250. Green-light irradiation reverses the diarylethenes back to the ring-open form, fully restoring the photoluminescence on-state. Our approach further enables reversible multi-level data storage and near-infrared anti-counterfeiting, highlighting the potential for smart light-responsive materials and optoelectronic devices.

## Abstract (web summary)

Achieving photoswitchable luminescence across a broad wavelength range remains challenging. Here, the authors integrate diarylethene photoswitches with quantum dots, enabling visible-light on-off switching of photoluminescence from blue to near-infrared across nearly 500 nm range, with applications in data storage and anti-counterfeiting.

## Introduction

Light-responsive materials, which exhibit distinct physical and chemical properties in response to the non-invasive light stimuli, have received significant attention in fields such as data processing^1,2^, biological science^3,4^, and smart optoelectronic devices^5–7^. The integration of on/off switching functionality is highly attractive for next-generation smart light-responsive materials, which are typically achieved by combining strong emissive materials with molecular photoswitches. Quantum dots (QDs), an ideal class of photoluminescence (PL) nanomaterials, possess broadband absorption^8^, narrow emission^9^, and size-tunable bandgap due to the strong quantum confinement effect^10,11^. They have been widely applied in bioimaging^12,13^, display^14,15^, and quantum photonic technology^16,17^, and further coupling of PL on/off switching could further expand their potential in high-resolution imaging, optical memory, and encryption. Molecular photoswitches, such as diarylethene (DAE), azobenzene, spiropyran (SP), can reversibly interconvert between two stable isomeric states upon light irradiation at different wavelengths. When coupled with QDs, only one isomer can effectively quench the QD’s PL via energy transfer or charge transfer, representing the PL off-state, while the other isomer, exhibiting no or weak interaction with QD, maintains the PL on-state. Therefore, combining QDs with molecular photoswitches provides a versatile strategy for constructing advanced light-responsive materials with dynamically tunable optical functionalities.

However, to date, all reported QD-photoswitch systems modulate only a single type of QD within a narrow spectral range due to the requirement of matched energy levels and/or spectral overlap. For instance, SP photoswitches and DAEs were employed to switch the green PL of CdSe/ZnS QDs through Förster resonance energy transfer (FRET) from QDs to the merocyanine or closed-form DAEs^18,19^, CdSe QDs functionalized with furylfulgide photoswitches exhibited red PL on/off switching via a photoinduced electron transfer process^20^, and DAEs anchored to PbS QDs enabled the near-infrared (NIR) PL switch through triplet energy transfer (TET)^21^. It is highly challenging to develop a general strategy for PL on/off switching of various QDs spanning a broad spectral range. Moreover, current on/off switching of QDs' PL relies on the use of UV light to trigger the photoisomerization of the photoswitches. The UV light with limited penetration depth can induce by-product formation and cause photooxidation, thereby restricting further applications in biological and optoelectronic systems^22,23^. Therefore, it is highly desirable to develop QDs with PL on/off switching functions that can be operated under visible-light activation. Many research efforts have contributed to developing all-visible-light activated molecular photoswitches via extending π-conjugation^24^, employing triplet sensitization^25^, or integrating upconversion nanoparticles^26^. For example, charge-transfer complex sensitizers were utilized to achieve bidirectional visible-light activated DAEs via triplet sensitization^27^. The rational design of perylene bisimide-based photoswitches enabled long-wavelength responsiveness with robust thermal bistability through triplet-mediated pathways^28^. However, their use in reversible on/off PL switching of emissive materials remains largely unexplored. Realizing such functionality demands molecular photoswitches that can operate efficiently under visible light with good reversibility and simple compatibility with diverse PL materials. Meanwhile, it should preserve the intrinsic photophysical properties of the emissive materials in the PL on-state. Importantly, it must achieve precise and reproducible switching to the PL off-state over a broad wavelength range with high on-off contrast.

**Fig. 1:**
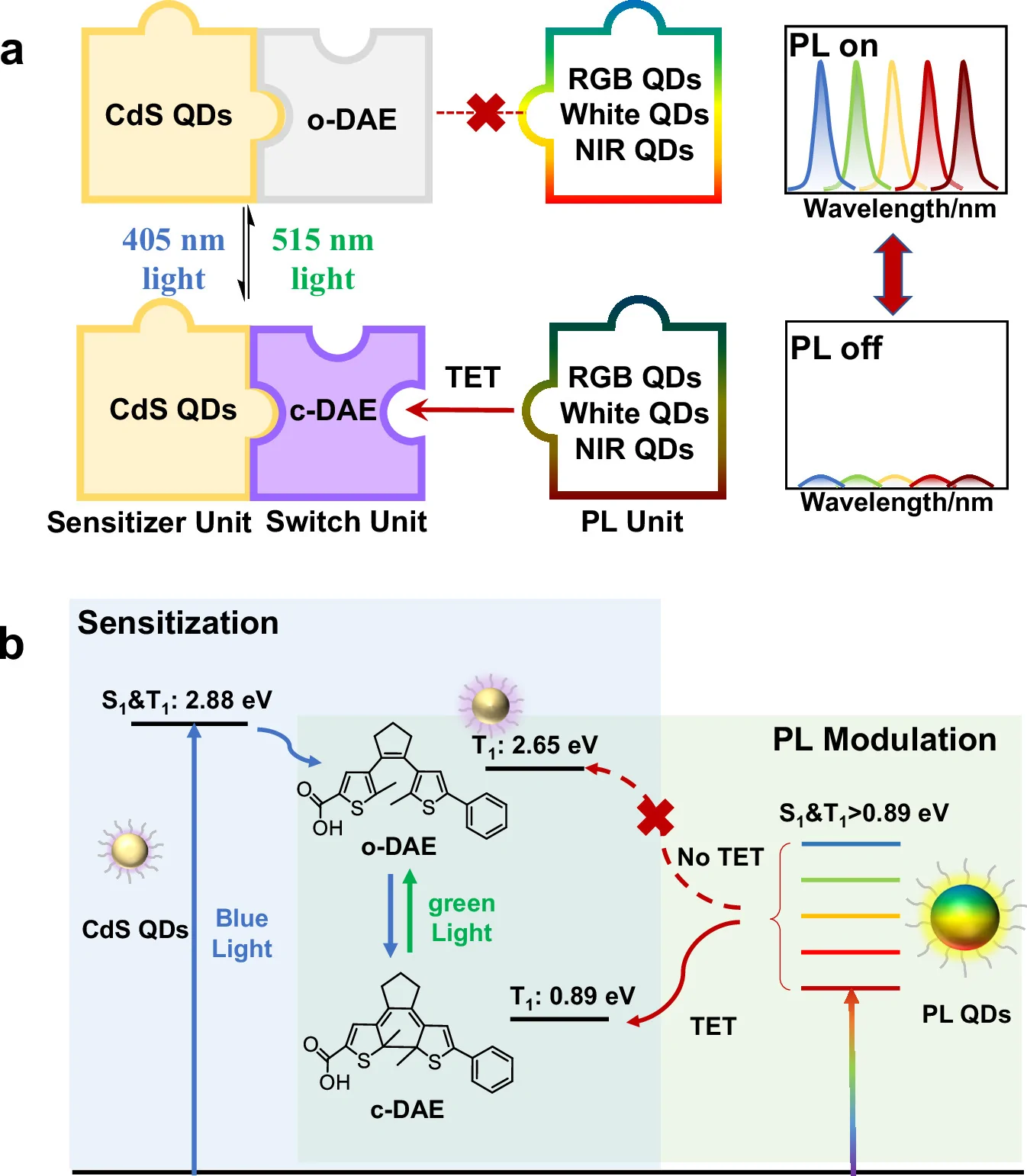
Schematic illustration of on/off switching of QDs emission across wide spectral range via all-visible-light-activated DAEs. **a** Reversible PL on/off switching of various QDs using CdS QDs sensitized DAEs upon by alternating 405 nm and 515 nm light irradiation. **b** Under blue light excitation, CdS QDs sensitize o-DAE through triplet energy transfer to form c-DAE, which allows to quench the PL of various QDs emitting from blue to NIR via TET; Subsequent green light irradiation converts c-DAE back to o-DAE, blocking the transfer pathway and restoring the PL of QDs to the on-state.

Here, we report an example of reversible on/off PL switching of various QDs across an ultrawide spectral range from 454 to 950 nm, enabled by visible-light activated DAEs through triplet sensitization from CdS QDs, (Fig. 1). The mechanism of our design relies on the distinct triplet excited energy levels (T_1_) of the two DAE isomers, 2.65 eV of o-DAE and 0.89 eV of c-DAE^21^, respectively. For QDs such as size-tuned CdZnSeS gradient alloy QDs (blue, green, or red emission), ZnCuGaInS@ZnS QDs (white emission), and PbS QDs NIR emission, whose excited-state energies lie between the large T_1_ energy gap of the two DAE isomers, the PL of these QDs can be reversibly modulated between on- and off-state. No TET occurs from QDs to the o-DAE, but significant TET with unity efficiency to c-DAE, yielding PL on/off ratios up to 250. All-visible-light reversible switching between the o-DAE and c-DAE was achieved through triplet sensitization from CdS QDs with the T_1_ energy higher than that of o-DAE. Therefore, the photocyclization can proceed efficiently via the low-lying triplet excited state under 405 nm irradiation, replacing the conventionally used UV light. The back switching from c-DAE to o-DAE occurs via the singlet excited state upon 515 nm green light irradiation. Notably, our strategy relies on a simple energy alignment between the DAE and various QDs, the latter of which can be readily tuned through simple size control to the desired wavelength. Moreover, we demonstrate that reversible multi-level data storage and information encryption can be realized through PL on/off switching in the NIR region, highlighting the potential of all-visible light-controlled responsive materials for advanced applications in photoelectronic and information processing.

## Results

### Modulation behavior

**Fig. 2:**
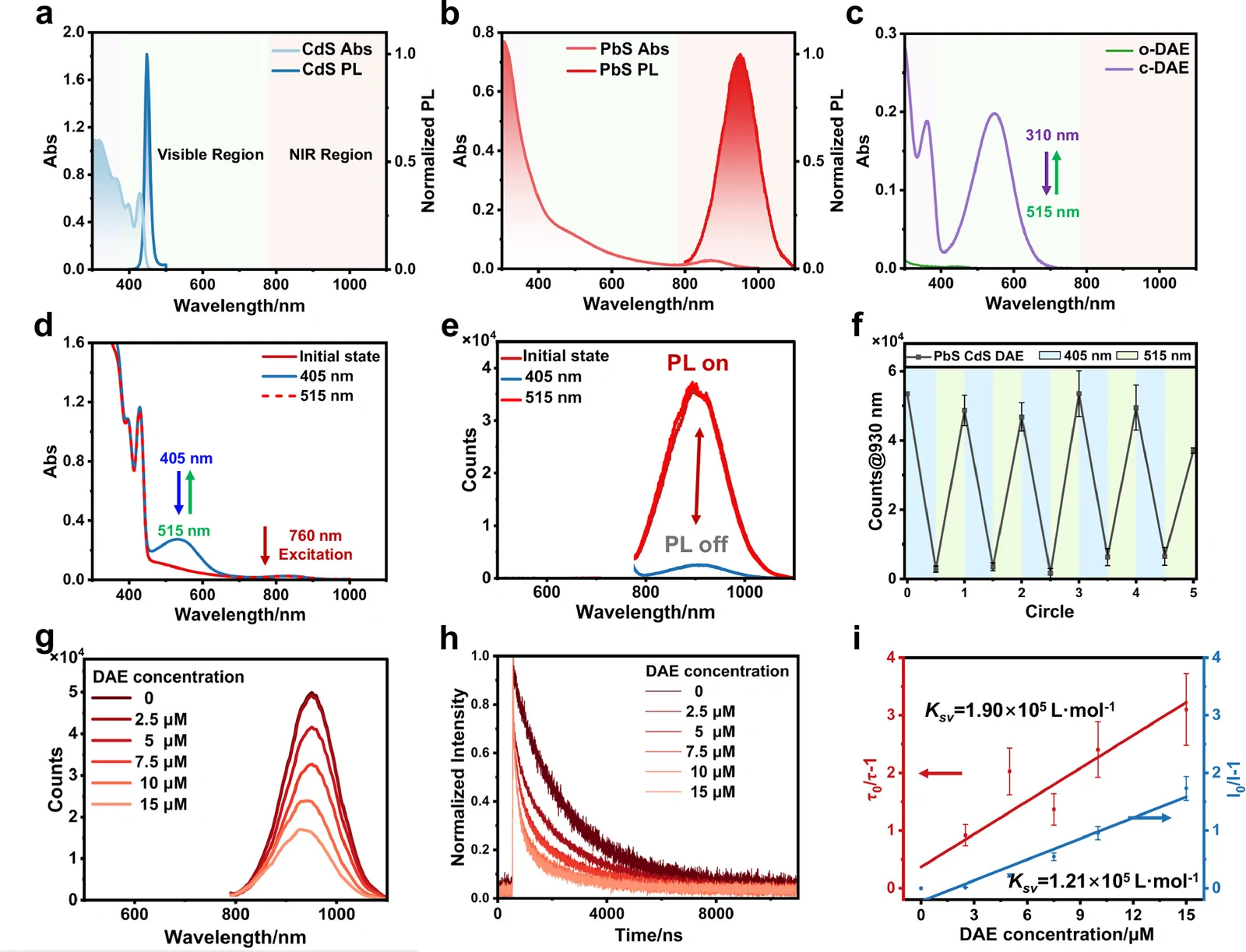
Photophysical properties of the CdS QDs-DAE-PbS QDs system. Absorption and PL spectra of **a** 5 µM CdS QDs and **b** 2 µM PbS QDs. **c** Absorption spectra of 100 μM DAE converted between o-DAE and c-DAE isomers upon UV and green light irradiation. **d** Absorption spectra and **e** PL spectra of the sample containing 8 µM CdS QDs, 160 µM DAE and 2 µM PbS QDs under 405 nm and 515 nm light irradiation. **f** Reversible PL modulation of PbS QDs demonstrated by the intensity at 930 nm over five cycles of alternating 405 nm and 515 nm light irradiation. Error bars represent the standard deviation of three independent measurements (*n* = 3). **g** Steady-state PL spectra and **h** time-resolved PL decays of 2 µM PbS QDs with increasing c-DAE concentration (0, 2.5, 5, 7.5, 10, and 15 µM). **i** Stern-Volmer plot of PL intensity quenching and lifetime quenching. All the samples were prepared in toluene, and the PL spectra in the NIR range have been corrected. Error bars represent the relative standard error of the fitted slope. Source data are provided as a Source Data file.

To demonstrate PL on/off switching via all-visible-light control in our design, PbS QDs that emit in the NIR region were first examined. The PbS QDs and CdS QDs used for DAE sensitization were synthesized according to previous reports^29,30^. The absorption and PL spectra of CdS QDs and PbS QDs in toluene are shown in Fig. 2a, b. CdS QDs display the first excitonic absorption peak at 430 nm and emit in the visible region with a maximum at 450 nm. The first excited-state energy of CdS QDs, characterized by strong spin-orbit coupling, was determined to be 2.88 eV from the first excitonic band. With an excited-state energy higher than the T_1_ level of o-DAE, the synthesized CdS QDs enable triplet-sensitized photocyclization to c-DAE, as we have previously reported^31^. PbS QDs exhibit an absorption peak at 875 nm and emit in the NIR region with a maximum at 950 nm. The first excited state energy of PbS QDs is determined to be 1.42 eV, lying between the T_1_ levels of o-DAE and c-DAE. The transmission electron microscopy (TEM) images show that both CdS QDs and PbS QDs feature highly uniform morphology, with an average size of 2.9 nm for CdS QDs and 3.0 nm for PbS QDs (see Supplementary Fig. 1 and Supplementary Fig. 2). Figure 2c presents the absorption spectra of o-DAE and c-DAE isomers. Upon irradiation with 310 nm UV light (see all the entire light irradiation process in the Methods), o-DAE undergoes photoisomerization via the singlet excited state surface to form the c-DAE isomer^32^, characterized by the appearance of a new absorption band centered at 545 nm. Subsequent exposure to 515 nm light of c-DAE induces full recovery to the o-DAE isomer, indicating the reversibility of the photoisomerization process. Notably, irradiation at 405 nm of the solution containing only o-DAE cannot induce photoisomerization, as the molecules do not absorb in this region and the photon energy is too low to access the singlet state.

To illustrate our strategy with the all-visible light-activated switching process, a solution containing 8 µM CdS QDs, 160 µM o-DAE and 2 µM PbS QDs was prepared in deaerated toluene via direct mixing, and the corresponding absorption spectra are shown in Fig. 2d. The carboxylic acid group of the DAE molecules enable dynamic and reversible anchoring onto the surfaces of both CdS QDs and PbS QDs, thereby facilitating highly efficient Dexter-type energy transfer. The absorption spectrum of the mixed solution displays the combined spectral features of CdS QDs, DAE, and PbS QDs, confirming that these three components exhibit very weak interactions in their ground states. Upon 405 nm light irradiation of the mixed solution, the typical absorption band of c-DAE in the visible region appears, consistent with the observation under direct UV light irradiation of o-DAE. Notably, since only a small amount of CdS QDs was added to the solution, at a molar ratio 1:20 relative to DAEs, the photostationary state (PSS) upon 405 nm irradiation consisted of 90% c-DAE and 10% o-DAE, identical to that obtained via direct UV-induced photocyclization of DAE^21,31^. Subsequent green light irradiation, the absorption spectrum of the mixed solution fully recovery to the initial state, indicating the efficient and reversible photoisomerization of DAEs. It should be noted that the presence of small amounts of PbS QDs in the solution does not affect the isomerization of DAEs under all-visible-light control.

The key to facilitating efficient all-visible-light-controlled photoswitching in our design is through two-step TET from CdS QDs and PbS QDs to DAEs. To evidence the TET processes from QDs to DAE, femtosecond transient absorption spectroscopy (fsTA) was performed, and the spectra are shown in Supplementary Figs. 3, 4. The CdS QDs display an excited-state absorption (ESA) around 385 nm and a ground-state bleach (GSB) around 430 nm. After introducing o-DAE, the transient signal of CdS QDs changes notably faster. Specifically, the GSB signal recovered to 55.0% of its initial amplitude at 7 ns for bare CdS QDs, whereas only 9.5% of the initial bleach remained in the CdS QD/o-DAE mixture. No new spectral features are observed within the time window of our pump-probe delay stage (7 ns), suggesting the absence of significant electron transfer. FRET from CdS QDs to DAE can also be ruled out, as there is no overlap between the absorption spectra of o-DAE and the PL spectrum of CdS QDs. The TET process between PbS QDs and DAE was also probed. Compared with bare PbS QDs and the PbS QD/o-DAE mixture, the PbS QD/c-DAE mixture exhibits accelerated recovery of GSB signals of PbS QDs around 875 nm. At 7 ns, the GSB signal retained 64.8% and 63.2% of its initial amplitude for bare PbS QDs and the PbS QD/o-DAE mixture, respectively, whereas only 52.0% remained for the PbS QD/c-DAE mixture. Two transient absorption bands at 488 and 560 nm emerge during the decay of the excited state of PbS QDs (Supplementary Fig. 4), which is consistent with the observation of triplet c-DAE formation in our previous report^21^. Therefore, we conclude that TET process from QDs to c-DAE is the dominant mechanism responsible for both the sensitization of DAE photoisomerization and the reversible PL modulation process. Previous studies on QD sensitization of triplet molecules indicate that the efficient TET between QDs and organic molecules depends on donor-acceptor wavefunction overlap^33–35^. Therefore, it is essential to ensure close proximity between QDs and DAE molecules, which is achieved by introducing the carboxyl functional groups of DAEs to dynamically anchor to the surfaces of both types of QDs. The non-carboxyl functionalized DAEs were also mixed with CdS QDs and PbS QDs, and the molecular structure, absorption and PL spectra of the mixed solutions are shown in Supplementary Fig. 5. Under identical 405 nm irradiation conditions (the same intensity and duration used for carboxylated DAEs), no changes in the absorbance or PL spectra were observed, indicating the absence of ring-closed DAEs formation and that no efficient TET occurred between DAE and QDs in absence of anchoring groups. In addition, in the absence of CdS QDs, the PbS QD/DAE mixture shows no spectral signatures of DAE isomerization under 405 nm light irradiation (Supplementary Fig. 6), indicating that CdS QDs act as essential triplet sensitizers enabling the ring-closing photoisomerization of DAEs under visible-light excitation.

The capability of on/off switching of NIR PL was investigated during the all-visible-light activation of DAEs as illustrated in Fig. 2e. The PL excitation wavelength of PbS QDs was set to 760 nm, which is not absorbed by either o-DAE or c-DAE, demonstrating the orthogonal control over the switching function and PL readout in our design. When DAE is in the ring-open form, the NIR PL of PbS QDs remains in the on-state. The NIR PL peak of the solution is blue-shifted by 55 nm compared with that of the bare PbS QDs solution, which may be attributed to the modifications in the excited-state electronic environment of QDs induced by the anchored molecules^20^. Upon exposure of the mixed solution to 405 nm light for 90 seconds, the o-DAEs efficiently photoisomerize to the closed form through sensitization by CdS QDs. Meanwhile, the NIR PL of PbS QDs is significantly quenched to the PL off-state with an efficiency of 93.6%. The PL on/off ratio, which is determined as the ratio of the intensity peaks between the PL on and PL off states, was calculated to be 15.6 in this case. When subsequent irradiation with green light to the mixed solution, DAEs convert back to the ring-open form and the NIR PL of PbS QDs fully recovers to its original on-state. Therefore, our strategy demonstrates that the NIR PL of QDs can be reversibly modulated by using all-visible-light activated DAEs with high efficiency and orthogonal light operation. Importantly, the system exhibits good reversibility and fatigue resistance over five write-erase cycles (Fig. 2f), highlighting its potential for recording information. Notably, bare PbS QDs did not exhibit PL modulation behavior reversibly under alternative irradiation of 405 nm and 515 nm (Supplementary Fig. 7).

To investigate the energy transfer efficiency from PbS QDs to the visible-light activated DAEs, we performed steady-state and time-resolved PL measurements. The concentration of c-DAE in the mixed solution was controlled by adjusting the duration of 405 nm irradiation. With increasing concentration of c-DAE, remarkable reduction in both PL intensity and lifetime of PbS QDs are observed in Fig. 2g, h. With c-DAE concentrations of 2.5, 5, 7.5,10, and 15 µM, the PL intensity was quenched with an efficiency of 1.4%, 17.2%, 34.2%, 48.7% and 63.3%, and the average lifetime of PbS QDs decreased from 2.11 µs to 1.10 µs, 0.70 µs, 0.89 µs, 0.62 µs and 0.52 µs, respectively. Specifically, when the concentration ratio of PbS QDs to c-DAE reached 1:80, the PL average lifetime decreased significantly to 0.12 µs (Supplementary Fig. 8). Stern-Volmer analysis was also carried out using both PL intensity and lifetime data to gain deeper insight into the efficiency of the TET process from PbS QDs to c-DAEs, as depicted in Fig. 2i. The Stern-Volmer constants (*K*_*SV*_, detailed in the Methods), determined from the fitting are 1.21 × 10^5 ^L·mol^−1^ for PL intensity and 1.90 × 10^5 ^L·mol^−1^ for PL average lifetime, respectively. The efficient quenching process in our design is consistent with diffusion-controlled quenching process^36^, and also comparable to the reported PL modulation systems under UV light activation^18^.

**Fig. 3:**
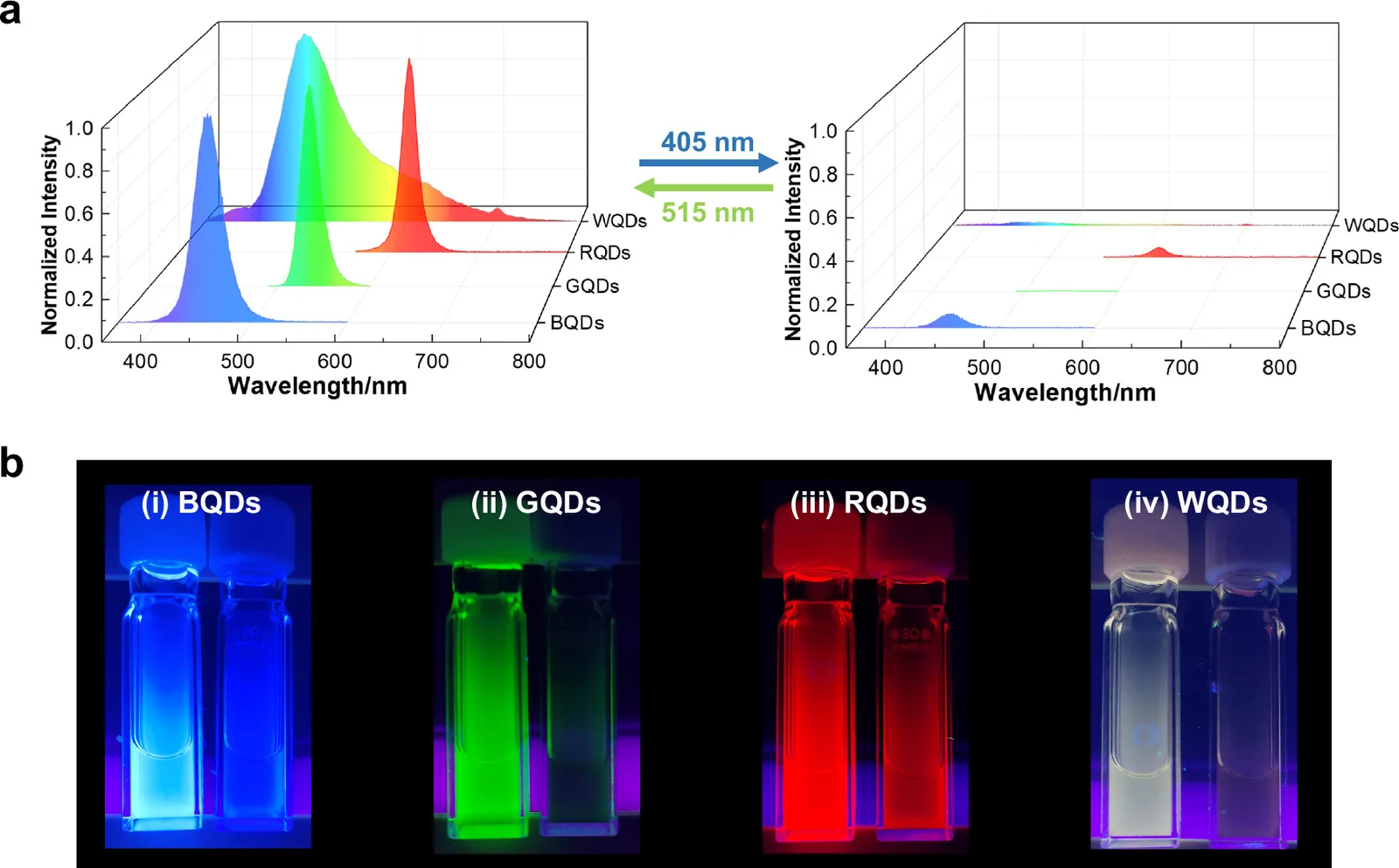
PL on/off switching across visible light region using CdS QDs sensitized DAEs. **a** Normalized PL spectra of QDs emitting blue, green, red, and broad white-color mixed with CdS QDs and DAEs, recorded before and after 405 nm irradiation. **b** Photographic images of the corresponding QD samples (i-iv) under UV lamp, showing distinct PL on and off-state before and after 405 nm light irradiation. For each pair of vials, the left contains PL QD/o-DAE/CdS QD mixtures and the right contains PL QD/c-DAE/CdS QD samples. Source data are provided as a Source Data file.

In principle, our strategy can use the visible-light activated DAEs to switch the PL on and off for any QDs whose excited state energies lie between the T_1_ levels of o-DAE and c-DAE, with the capability of extending beyond the NIR region to cover the entire visible spectrum. To demonstrate the general applicability, a series of QDs were synthesized (see the detailed synthesized in the Methods) with emission wavelengths covering the different visible band. These QDs include size-tuned CdZnSeS gradient alloy QDs emitting blue (454 nm, BQDs), green (534 nm, GQDs), and red (621 nm, RQDs) color, and ZnCuGaInS@ZnS QDs exhibiting broadband white color (360–794 nm, WQDs), see the absorption and PL spectra in Supplementary Fig. 9. Following the same approach used for NIR PL switching, these QDs were directly mixed with CdS QDs and o-DAEs, and the PL of all these QDs remain in the on-state as shown in Fig. 3a left. Upon exposure to 405 nm light for 90 s, DAEs undergo ring-closed isomerization (see the absorption spectra in Supplementary Fig. 10) and the PL intensities of all QDs are markedly quenched to the off-state with efficiencies of 93.0% for BQDs, 99.6% for GQDs, 95.1% for RQDs, and 97.6% for WQDs and PL on/off ratio of 14.3, 250, 20.4, 41.7, respectively. The quenching efficiency of our system in the PL off-state represents the highest observed, even compare to previously reported UV-light-activated PL modulation system operating in the visible region^37,38^. Further exposure to 515 nm green light, the PL spectra of all QDs recovered to the original on-states. Figure 3b shows the corresponding PL photographic images of these QDs solution before and after 405 nm irradiation under 365 nm UV lamp. It should be noted that the intensity of 365 nm is very weak, which is insufficient to induce obvious photoisomerization of the DAEs during measurement due to the low absorbance at this wavelength (Fig. 2c). A remarkable contrast can be observed between the bright on-state when DAEs are in the ring-open form and the significantly diminished PL to the off-state when DAEs switched to the ring-closed form upon 405 nm irradiation. The comparison with previously reported switchable QD systems under UV-light activation is summarized in Supplementary Table 3. The key performance parameters, such as PL wavelength range, activation light, PL on/off ratio, fatigue resistance, etc., are included in the table. Our strategy represents an efficient approach to reversibly switching the PL of emissive materials using all-visible-light-activated molecular photoswitches. Moreover, it can efficiently modulate emission across such a broad spectral range from blue to the NIR region. These capabilities further inspire us to investigate the potential for advanced optical applications, particularly in areas such as multi-level data storage and anti-counterfeiting.

### Multi-level data storage

**Fig. 4:**
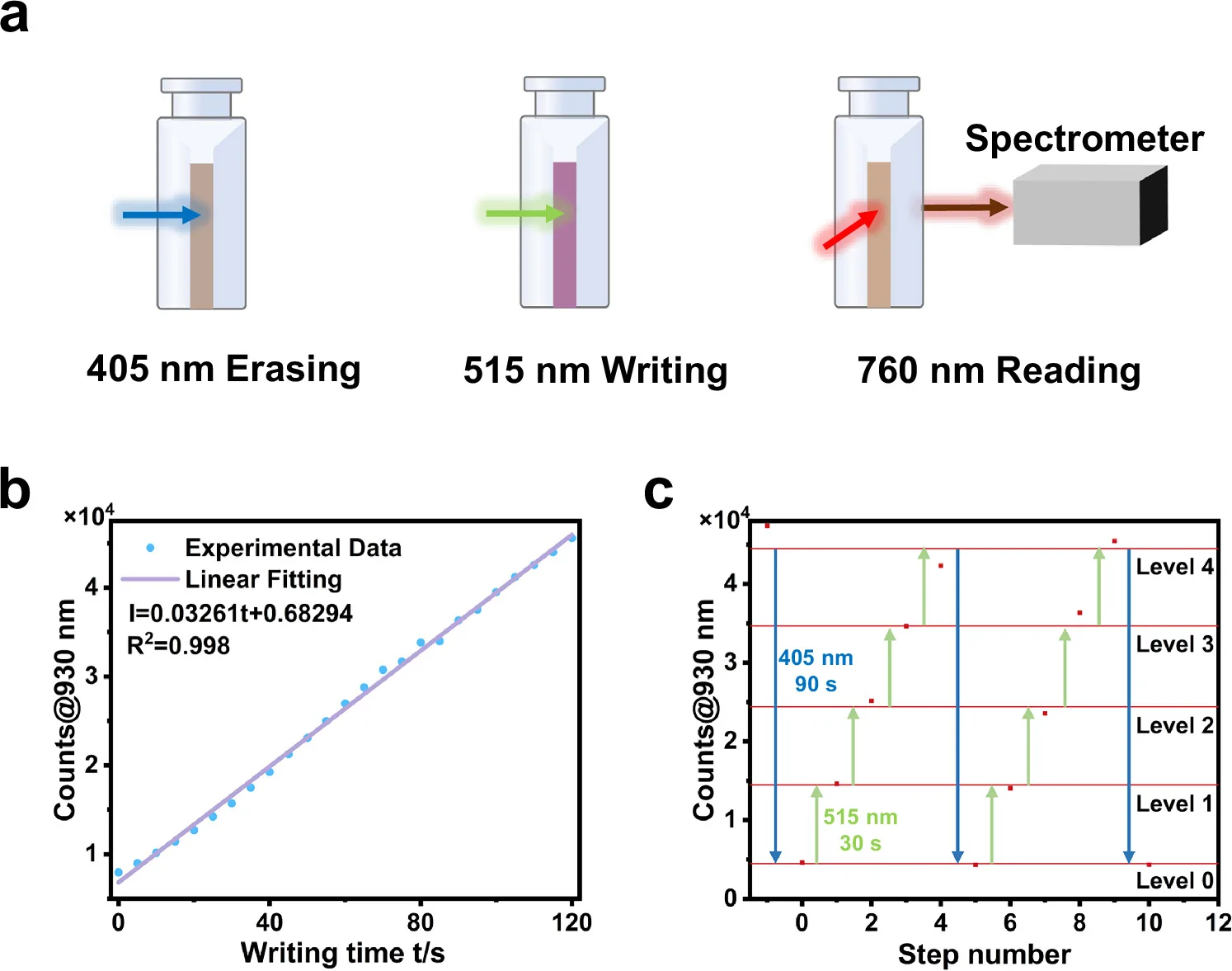
Reversible optical multi-level data storage based on PbS QD/DAE/CdS QD system. **a** The schematic process of the multi-level optical storage. The initial PL off-state was set with 405 nm light irradiation, the information was written with 515 nm light at a step of 5 s, and the stored information was read under 760 nm excitation with the output NIR PL at 930 nm. **b** The experimental PL intensity data is fitted to a linear function with 24 irradiation steps. **c** The signal was divided into 4 levels by an interval of 10,000 photon counts defined as a single quantization level and the corresponding writing time for each signal level are set to 30 s. Source data are provided as a Source Data file.

The high contrast between the PL on and off-states in our design enables its applications in optical data storage. Leveraging the orthogonal light control of the PbS QD/DAE/CdS QD system, the optical memory functionality can be achieved as shown in Fig. 4a using 515 nm light for writing, 405 nm light for erasing, 760 nm excitation for nondestructive reading, and the PL intensity of PbS QDs at 930 nm serving as the output signal. Multi-level optical memory requires fine-tuning of each PL state with a high number of distinguishable intensity levels. In our system, the output PL intensity is correlated to the fraction of DAE molecules converted to the closed form, which can be finely tuned through the irradiation dose (intensity × time). The initial PL off-state was first set by 405 nm irradiation (19.10 mW/cm^2^) for 2 min to define the baseline and erase residual background emission prior to multi-level data encoding. The memory was then written by exposed to a 515 nm light (198 mW/cm^2^) at step of 5 s for 24 steps, leading to a progressive PL intensity increase controlled by the number of irradiation steps. Each irradiation step generated a ΔPL intensity increase of ~1580 counts, due to the reducing number of c-DAEs. The optical output can be quantitatively converted into a linear, multi-staged digital signal as shown in Fig. 4b. If an interval of 10,000 photon counts was defined as a single quantization level, a reversible optical memory with a data storage capacity of 4 PL intensity levels can be achieved as depicted in Fig. 4c. Subsequent irradiation with 405 nm light, the stored memory can be fully erased and ready for writing new information. To evaluate the long-term stability of the photoswitching and memory functions towards practical applications, the PL intensities of PbS QD/DAE/CdS QD at three distinct memory states were monitored over 7 days under ambient atmosphere when stored in the dark at 24 °C and 25% relative humidity. As shown in Supplementary Fig. 11, the PL intensities of the on, level-2, and off states remain nearly constant throughout the storage period, demonstrating the excellent thermal stability of the memory states of our design under ambient conditions.

We also examined the memory functionality in the visible-emitting range, employing GQD/DAE/CdS QDs as a representative system for demonstration. The optical memory functionality was demonstrated using 550 nm light (104 mW/cm^2^) for writing, 405 nm light for erasing, 480 nm excitation (0.38 mW/cm^2^) for nondestructive reading, and the PL intensity of GQDs at 530 nm serving as the output signal. When an interval of 117 a.u. photon counts at 530 nm was defined as a single quantization level, a reversible green-emitting memory can be achieved with a data storage capacity of 4 levels (Supplementary Fig. 12a). Notably, GQD/DAE/CdS QD also exhibits excellent memory stability over 7 days storage (Supplementary Fig. 12b). Furthermore, after depositing GQD/DAE/CdS QD with polystyrene polymer onto an ITO substrate, the optical memory functionality was still retained in the solid state even after 14 days of storage (Supplementary Fig. 13), highlighting the potential for future optoelectronic data storage applications. Future employing pulsed light source and reducing the power of incident light can largely enhance the memory levels.

### NIR anti-counterfeiting

**Fig. 5:**
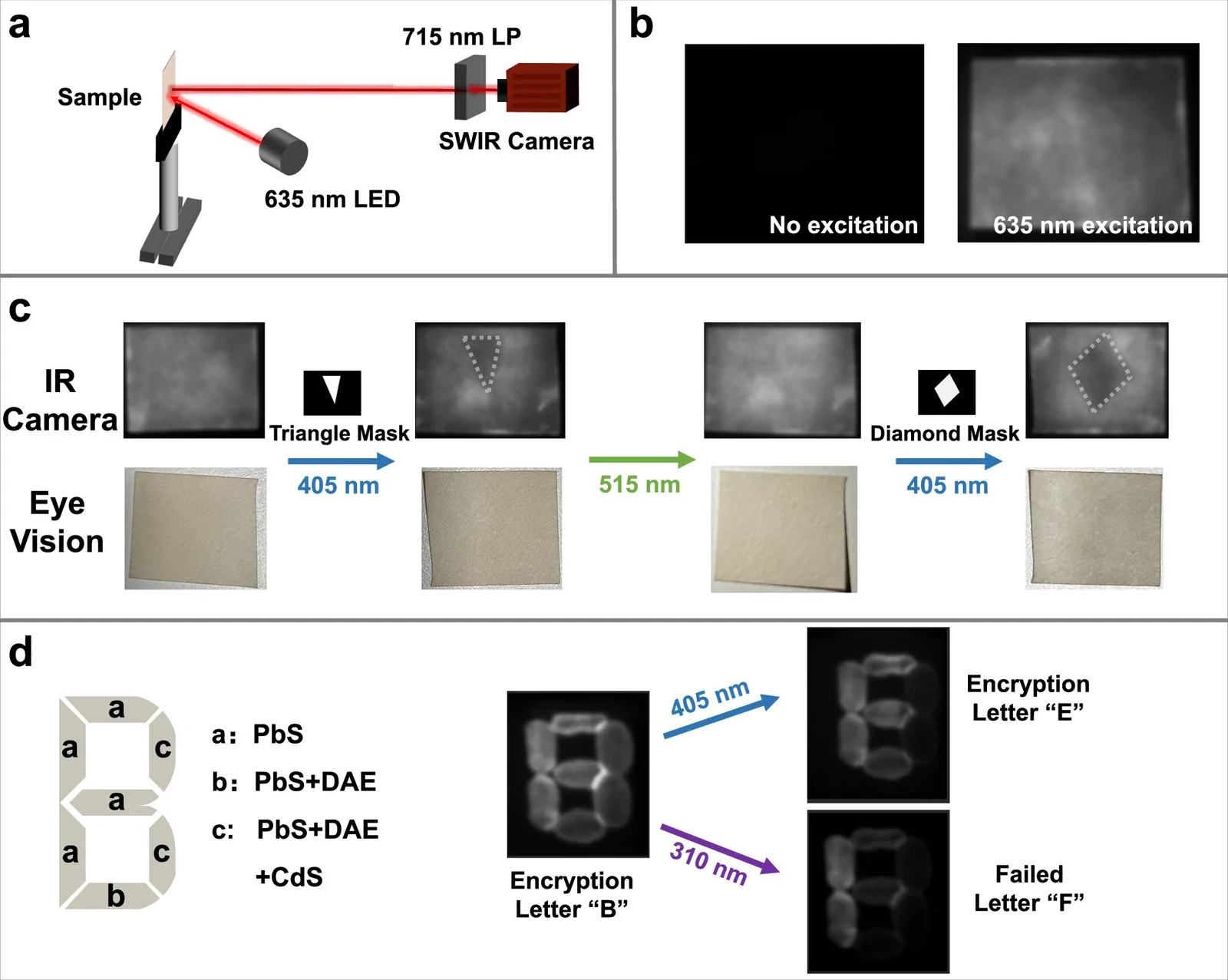
Schematic and experimental setup for optical information encryption and decryption. **a** The information read setup consists of a 635 nm LED for excitation and an IR camera for imaging. LP is 715 nm long pass filter. **b** IR Images of the sample without excitation (left) and with 635 nm excitation (right). **c** Reversible writing and erasing of triangle and diamond IR patterns visualized by the IR camera after exposure to 405 nm light followed by 515 nm light. The eye vision images were obtained using a standard phone camera for comparison. **d** Optical information encryption using region-specific deposition: **a** PbS QD, **b** PbS QD/DAE, and **c** PbS QD/DAE/CdS QD. The initial encrypted letter “B” transforms into decrypted letter with true information of “E” under 405 nm irradiation, and irradiation with 310 nm light led to the false information of “F”. Source data are provided as a Source Data file.

The above solid state performance and the modulation of invisible NIR PL with enhanced confidentiality highlights their significant potential for high-security optical information storage and encryption applications. To prove the concept, we prepared a solid-state sample by depositing a mixed solution of CdS QDs (400 μM), o-DAE (2 mM), and PbS QDs (20 μM) onto a 30 × 30 mm filter paper, which was utilized for reversible write-erase NIR pattern demonstrations. As shown in Fig. 5a, the encrypted information of the sample is readable only with an IR camera under red LED excitation (such as at 635 nm). In the absence of excitation, the deposited sample exhibited no IR signal in dark, whereas under 635 nm illumination, an IR image of the paper can be clearly detected as illustrated in Fig. 5b. When alternative irradiation with 405 and 515 nm light to this paper with the masks, various written and erased patterns can be visualized under the IR camera (Fig. 5c). Specifically, when the sample exposure to 405 nm light through a triangle mask, the PL of PbS QDs in the unmasked triangle region was quenched, resulting in the appearance of a triangular pattern visualized by the camera. Subsequent exposure to 515 nm light, the quenched regions switched back to the PL on-state and fully erased the pattern. By introducing different masks, such as a diamond-shaped pattern, different patterns can be sequentially written on the same paper. Importantly, during these reversible write-erase steps, the sample exhibited no obvious color change or discernible pattern to the naked eye or a standard mobile phone camera, as illustrated at the bottom of Fig. 5c.

Our design was further employed to achieve advanced information encryption capabilities. As illustrated in Fig. 5d, an encryption pattern of an anti-counterfeiting digital number “B” was written by selectively depositing PbS QDs, PbS QD/DAE, and PbS QD/DAE/CdS QD in different regions. The as-prepared pattern was initially observed as the encrypted letter “B” under the IR camera. Upon 405 nm irradiation, the CdS QD-containing regions was selectively quenched, resulting in the conversion of the letter “B” into a decrypted letter “E” for the presentation of true information. In contrast, direct irradiation with 310 nm light to letter “B” with all DAE-containing regions to the NIR PL off-state, leading to the appearance of letter “F” that corresponding to the false information. The distinct information under different wavelength light irradiation provides an additional control of security for anti-counterfeiting and confidential communication.

## Discussion

In conclusion, we develop a general strategy for all-visible-light activated on/off PL switching of QDs spanning an ultrawide spectral range from 454 to 950 nm, achieved via a simple direct-mixing approach combining CdS QDs, DAE photoswitches, and PL QDs. This approach enables efficient and fully reversible PL modulation under alternating 405 nm and 515 nm light irradiation, achieving quenching efficiencies up to 99.6% with the PL on/off ratio over 250. Taking advantage of the orthogonal light wavelength control, as well as the robustness and reversibility of our PL switching materials, we further demonstrated the application in multi-level optical data storage with 4 levels and anti-counterfeiting in the IR region. Furthermore, extending this strategy to heavy-metal-free QDs, such as ZnSe QD, InP QD or CuInSe₂ QD-based systems, may broaden the scope of biological applications. Overall, our work highlights a versatile strategy for constructing all-visible-light responsive materials, offering promising opportunities for smart optoelectronic devices and precise control in data processing.

## Methods

### Chemicals

Cadmium oxide (CdO, 99.99%), cadmium acetate (Cd(OAc)_2_, 99.99%), lead oxide (PbO, 99.999%), zinc oxide (ZnO, 99.99%), Zinc iodide (ZnI₂, 99.9%), zinc stearate (Zn(st)₂, 99.9%), zinc acetate (Zn(OAc)_2_, 99.99%), cuprous iodide (CuI, 99.99%), gallium acetylacetonate (Ga(acac)₃, 99.99%), indium iodide (InI_3_, 99.99%), selenium (Se, 99.99%), oleylamine (OLA, 97%), 1-dodecanethiol (DDT, 95%), oleic acid (OA, 90%), 1-octadecene (ODE, 90%), tri-*n*-octylphosphine (TOP, 90%), and 1-octanethiol (OT, 98.5%) were purchased from Shanghai Aladdin Chemical Reagent Co., Ltd., China. Bis(trimethylsilyl)sulfide ((TMS)_2_S, 95%) was purchased from Shanghai Macklin Biochemical Technology Co., Ltd., China. Sulfur (S, 99.98%) was purchased from Aldrich. Acetone and toluene were purchased from YuanLi, Tianjin. All the chemicals were used without further purification steps. Carboxyl (5-methyl-4-[2-(2-methyl-5-phenylthiophen-3-yl)cyclopenten-1-yl]thiophene-2-carboxylic acid) and non -carboxyl (3-[3,3,4,4,5,5-hexafluoro-2-(2-methyl-5-phenylthiophen-3-yl)cyclopenten-1-yl]−2-methyl-5-phenylthiophene) DAE were synthesized as reports^21,39^.

### Synthesis of QDs

#### CdS QDs

The synthesis of CdS QDs was modified slightly, referred to previous reports^29^. 257 mg of CdO, 6 mL of OA, and 16 mL of ODE were mixed in a 100 mL three-necked flask, and degassed under vacuum at 150 °C for 1 h. Then, the solution was heated to approximately 230 °C under a nitrogen atmosphere until the mixture turned clear and colorless. The sulfur precursor was prepared by dissolving 32 mg of sulfur in 3 mL of ODE degassed under nitrogen for 30 min, and then heated to 200 °C until fully dissolved. The sulfur solution was then injected into the cadmium precursor at 200 °C and reacted for 2 min. After injection, the reaction mixture was rapidly cooled by compressed air, reducing the temperature to 170 °C. The subsequent growth of CdS QDs was monitored via UV/vis absorption spectra. Once the absorption peak of CdS QDs reached 430 nm, the reaction mixture was cooled to room temperature and further purified by precipitation with acetone three times.

#### PbS QDs

PbS QDs were synthesized with slight modification according to previous reports^30^. 900 mg of PbO, 3 mL of OA and 10 mL of ODE were mixed in a 100 mL three-necked flask, which was degassed under vacuum at 120 °C for 1 h. After that, the solution was cooled down to 90 °C under N_2_. The sulfur precursor was prepared by dissolving 420 μL of (TMS)_2_S in 20 mL of ODE and degassing under N_2_ for 1 hour at room temperature. The sulfur precursor was then injected into the lead oleate solution at 95 °C for 2 min. After the reaction, the flask was quickly moved to the ice-water bath for cooling. When the flask reached room temperature, the obtained PbS QDs were further purified via precipitation with 60 mL acetone and centrifuged at 8000 r/min for 3 min. This purification was repeated twice additionally.

#### Blue-emitting CdSeS QDs

A precursor mixture containing CdO (1.0 mmol), Zn(OAc)_2_ (9.0 mmol), OA (8 mL), and ODE (15 mL) was first heated at 120 °C for 20 min under vacuum to eliminate residual moisture and oxygen. The solution was then switched to a nitrogen atmosphere and heated to 300 °C, at which point a mixture of ODE (3.0 mL) containing S (1.8 mmol) and Se (0.2 mmol) was swiftly injected. Following a 10 min nucleation period, a TOP-S solution (4 mL TOP with 8 mmol S) was added dropwise. After maintaining the reaction at 300 °C for 50 min to reach a steady state, the precursors—Zn(OAc)_2_ (2.5 mmol), ODE (4 mL), OA (1 mL), and OT (1 mL)—were slowly introduced, and the mixture was allowed to react for an additional 10 min before the annealing step.

#### Green-emitting CdSeS QDs

A precursor mixture containing Cd(OAc)_2_ (0.14 mmol), ZnO (3.42 mmol), OA (7 mL), and ODE (15 mL) was degassed at 120 °C for 20 min to eliminate water and oxygen. The solution was then placed under nitrogen and heated to 310 °C, followed by rapid injection of a TOP-based chalcogen solution (TOP 2.0 mL, S 2.0 mmol, Se 2.0 mmol). After 10 min of nucleation, an ODE-S solution (ODE 2.4 mL, S 1.6 mmol) was quickly added. 12 min later, the zinc precursor solution (Zn(OAc)_2_ 2.86 mmol, OA 1 mL, ODE 4 mL) was slowly introduced. When the temperature decreased to 270 °C, a TOP-S mixture (S 10 mmol, TOP 5 mL) was added dropwise, and the reaction proceeded for another 20 min before the annealing step.

#### Red-emitting CdSeS QDs

A mixture of CdO (0.16 mmol), Zn(OAc)_2_ (6 mmol), OA (7 mL), and ODE (15 mL) was degassed at 120 °C for 20 min. The solution was then heated to 300 °C under nitrogen, and a TOP-Se precursor (0.4 mL TOP with 0.8 mmol Se) was rapidly injected. After 15 min of growth, a second precursor solution (Se, 0.8 mmol; S, 0.2 mmol; TOP, 0.5 mL) was slowly added at 300 °C. The reaction was maintained for 30 min before a mixture containing S (0.8 mmol in 0.4 mL TOP) and CdO (0.3 mmol in 2 mL OA) was injected over 60 min. Subsequently, TOP-S (0.8 mmol S in 0.4 mL TOP) was introduced slowly, and the reaction was continued for another 30 min prior to annealing.

#### White-emitting ZnCuGaInS@ZnS

A mixture containing 1.5 mmol of ZnI_2_, 0.0625 mmol of CuI, 0.5 mmol of Ga(acac)_3_, 1 mmol of S, 0.05 mmol of InI_3_, 5 mL of OLA, and 1.5 mL of DDT was loaded into a three-neck flask and degassed at 80 °C under vacuum for 30 min to remove water and oxygen. The atmosphere was then switched to nitrogen, and the reaction temperature was raised to 240 °C for 5 min. 8 mmol Zn(OAc)_2_ dissolved in 8 mL OA and 4 mL ODE was rapidly injected and allowed to react for 75 min. Subsequently, 4 mmol Zn(OAc)_2_ dissolved in a mixture of 4 mL OA, 2 mL ODE, and 2 mL DDT was quickly added and the reaction was maintained at 240 °C for an additional 30 min. Finally, 4 mmol Zn(st)_2_ dissolved in 4 mL ODE and 2 mL DDT was injected, and the temperature was increased to 250 °C for a 60 min reaction, followed by natural cooling to room temperature.

### Sample preparation

All samples were prepared in a N_2_ glove box to ensure an oxygen-free atmosphere. The photos in Fig. 3b and the absorption spectra in Supplementary Fig. 10 were carried out in a 10 mm path quartz cuvette, and all the other tests were conducted using a quartz cuvette with a 2 mm path length. CdS QDs, DAE, and PL QDs (10 mg/mL CdZnSe@ZnSeS@ZnS QDs, 20 mg/mL ZnCuGaInS@ZnS QDs, and PbS QDs, respectively) were directly mixed in the toluene. The sample used for multi-level storage demonstration consisted of a PbS QD/DAE/CdS QD mixture with a molar ratio of 1:80:4. Polystyrene was used as a film-forming polymer matrix. and mixed with GQD/DAE/PS/CdS QD solution (mass ratio = 1:0.2:1:5). The mixture was deposited onto an ITO substrate via dynamic spin coating at 2000 rpm for 40 s to form thin films for data storage. For the solid-state anti-counterfeiting sample, a mixed solution containing 400 µM CdS QDs, 2 mM o-DAE, and 20 µM PbS QDs was deposited onto a 30 × 30 mm piece of filter paper.

### Molar absorption coefficient determination of QDs

The concentrations of CdS QDs were determined from absorption spectra using the molar extinction coefficient according to the empirical functions^40^:

1 $$D=(-6.6521\times {10}^{-8}){\lambda }^{3}+(1.9557\times {10}^{-4}){\lambda }^{2}-(9.2353\times {10}^{-2})\lambda+13.29$$

2 $$\varepsilon=21536{(D)}^{2.3}$$

where λ is the excitonic peak in the UV-visible absorption spectra, *D* is the diameter of

QDs and *ε* is the molar extinction coefficient at the absorption peak.

The concentrations of PbS QDs were determined from absorption spectra using the molar extinction coefficient according to the empirical functions^41^:

3 $$D=\frac{-0.283+\sqrt{0.080089+\frac{0.1008}{\frac{1240.8}{\lambda }-0.41}}}{0.0504}$$

4 $${\varepsilon }_{400}=0.0233{(D)}^{3}$$

where λ is the excitonic peak in the UV-visible absorption spectra, *D* is the diameter of PbS QDs and *ε*_400_ is the molar extinction coefficient at the absorption peak of 400 nm.

### Characterizations

UV-visible absorption spectra were collected using a Cary 60 UV-Visible spectrophotometer (Agilent). Visible PL spectra were recorded with a Cary Eclipse fluorescence spectrophotometer (Agilent), and NIR PL spectra were obtained using a fiber-coupled high-resolution spectrometer (HR-6XR300-25, Ocean Optics). The morphology of QDs was characterized by transmission electron microscopy (TEM, Tecnai G2 F20). The fsTA measurement was carried out (TA-100, Time-Tech Spectra, Co., Ltd.) using a Pharos2 fs-laser (Light Conversion; full width at half-maximum, ~222 fs, 100 kHz, 1030 nm), which was split into the pump and probe beams. The pump beam was directed into an optical parametric amplifier (OPA, Orpheus-HP, Light Conversion) to generate excitation pulses at 400 nm (1.2 nJ per pulse) or 780 nm (4 nJ per pulse), and was modulated at 500 Hz using an optical chopper for alternating pump-on and pump-off measurements. A broadband white-light continuum covering 380-600 nm (0.03 nJ per pulse) was generated by focusing a frequency-doubled 515 nm beam onto a sapphire plate. For the 500–900 nm probe range, the white-light continuum (0.05 nJ per pulse) was generated using a YAG crystal and filtered through a 900 nm low-pass filter. An optical delay stage (with maximum delay 7 ns) was used. The pump and probe beams were focused and overlapped on the sample in a 1 mm quartz cuvette. The transient spectra and kinetics were acquired by averaging 2000 measurements at every given time delay.

PL lifetime measurements of PbS QDs were carried out on a transient absorption spectrometer (LP980, Edinburgh Instruments) using an Nd:YAG laser (Agilite, Amplitude Continuum) equipped with an OPO (Horizon, Amplitude Continuum) generating a 10 ns pump beam and time-resolved traces were determined by a 5-stage PMT (R13456P, Hamamatsu) coupled with a monochromator (TMS302, Edinburgh Instruments) and recorded on an oscilloscope (RTM3002, Rohde and Schwarz). The NIR images were observed with an IR camera (Shanghai Boye Biotechnology Co., Ltd.) with a detection range of 900–1700 nm.

### Light sources

310 nm irradiation (1.77 mW/cm^2^) was performed using a UV analytic lamp. Visible light irradiation at 405 nm (19.10 mW/cm^2^) was carried out by using a continuous-wave laser (MDL-III-405). 515 nm irradiation (198 mW/cm^2^), 550 nm irradiation (104 mW/cm^2^), 635 nm irradiation (245 mW/cm^2^), and 760 nm irradiation (185 mW/cm^2^) were achieved using LED light sources from Zolix MLED 4-3. The multi-level storage experiments were carried out under the same irradiation conditions as described above.

### Calculation of Stern-Volmer constants

Stern-Volmer plots were further conducted for analysis the quenching of PL intensity (Eq. 5) and lifetime (Eq. 6):

5 $${{{{\rm{I}}}}}_{0}/{{{\rm{I}}}}=1+{K}_{SV}[Q]=1+{k}_{q}{\tau }_{0}[Q]$$

6 $${\tau }_{0}/\tau=1+{K}_{SV}[Q]=1+{k}_{q}{\tau }_{0}[Q]$$

where ${\tau }_{0}$ and τ are the average lifetime of PbS QDs without and with c-DAE, I_0_ and I are the PL intensity of PbS QDs without and with c-DAE, [*Q*] is the concentration of the c-DAE, *K*_*SV*_ is the Stern-Volmer constant, and the *k*_*q*_ is quenching rate constant.

## Supplementary information

Supplementary Information

Transparent Peer Review file

## Source data

Source Data

## Data Availability

The authors declare that all data supporting the findings of this study are provided in the paper and its Supplementary Information. Source data are provided with this paper.

## References

[1] Feng, Z. Y. et al. Dynamic multimodal information encryption combining programmable structural coloration and switchable circularly polarized luminescence. *Nat. Commun.* **16**, 2264 (2025). 10.1038/s41467-025-57649-1 PMC11885572 40050269

[2] Zhang, Y. F. et al. Ultraviolet irradiation-responsive dynamic ultralong organic phosphorescence in polymeric systems. *Nat. Commun.* **12**, 2297 (2021). 10.1038/s41467-021-22609-y PMC8052444 33863899

[3] Bi, S. H. et al. NIR-II responsive upconversion nanoprobe with simultaneously enhanced single-band red luminescence and phase/size control for bioimaging and photodynamic therapy. *Adv. Mater.* **35**, 2207038 (2023). 10.1002/adma.202207038 36398498

[4] Li, Z. Y. et al. A controllable photoresponsive potassium transporter. *Nat. Commun.* **16**, 6926 (2025). 10.1038/s41467-025-62113-1 PMC12304099 40721588

[5] Corrado, F. et al. Azobenzene-based optoelectronic transistors for neurohybrid building blocks. *Nat. Commun.* **14**, 6760 (2023). 10.1038/s41467-023-41083-2 PMC10622443 37919279

[6] Pan, T. et al. A flexible, multifunctional, optoelectronic anticounterfeiting device from high-performance organic light-emitting paper. *Light-Sci. Appl.* **11**, 59 (2022). 10.1038/s41377-022-00760-5 PMC8921225 35288540

[7] Xi, L. et al. Combining quantum dots and photochromic molecular switches: next-generation light-responsive materials. *Small Methods* **9**, 2500192 (2025). 10.1002/smtd.202500192 40357723

[8] Feng, Y. N. et al. Near-infrared light-activated Z-to-E isomerization of azobenzene triplet sensitization from PbS quantum dots. *Chem. Sci.* **16**, 16151–16157 (2025). 10.1039/d5sc03719k PMC12337389 40800058

[9] Wu, X. G. et al. Industry outlook of perovskite quantum dots for display applications. *Nat. Nanotechnol.* **17**, 813–816 (2022). 10.1038/s41565-022-01163-8 35869367

[10] Li, Y. M. et al. Stable and efficient CsPbI_3_ quantum-dot light-emitting diodes with strong quantum confinement. *Nat. Commun.* **15**, 5696 (2024). 10.1038/s41467-024-50022-8 PMC11228028 38972890

[11] Wise, F. W. Lead salt quantum dots: the limit of strong quantum confinement. *Acc. Chem. Res.* **33**, 773–780 (2000). 10.1021/ar970220q 11087314

[12] Chung, S. et al. Graphene quantum dots and their applications in bioimaging, biosensing, and therapy. *Adv. Mater.* **33**, 1904362 (2021). 10.1002/adma.201904362 PMC7289657 31833101

[13] Qu, D. et al. Tailoring color emissions from N-doped graphene quantum dots for bioimaging applications. *Light-Sci. Appl.* **4**, e364 (2015).

[14] Bian, Y. Y. et al. Efficient green InP-based QD-LED by controlling electron injection and leakage. *Nature* **635**, 854–859 (2024). 10.1038/s41586-024-08197-z 39567695

[15] Jia, Y. Y. et al. Long-range order enhance performance of patterned blue quantum dot light-emitting diodes. *Nat. Commun.* **16**, 7643 (2025). 10.1038/s41467-025-62345-1 PMC12357929 40819161

[16] Liu, S. F. et al. Quantum correlations of spontaneous two-photon emission from a quantum dot. *Nature* **643**, 1234–1239 (2025). 10.1038/s41586-025-09267-6 40634619

[17] Zou, M. et al. Realization of an untrusted intermediate relay architecture using a quantum dot single-photon source. *Nat. Phys.* **21**, 1670–1677 (2025).

[18] Zhu, L. Y. et al. Light-controlled molecular switches modulate nanocrystal fluorescence. *J. Am. Chem. Soc.* **127**, 8968–8970 (2005). 10.1021/ja0423421 PMC1435833 15969571

[19] Díaz, S. A. et al. Photoswitchable semiconductor nanocrystals with self-regulating photochromic Forster resonance energy transfer acceptors. *Nat. Commun.* **6**, 6036 (2015). 10.1038/ncomms7036 25592060

[20] Padgaonkar, S. et al. Light-triggered switching of quantum dot photoluminescence through excited-state electron transfer to surface-bound photochromic molecules. *Nano Lett.* **21**, 854–860 (2021). 10.1021/acs.nanolett.0c04611 33395307

[21] Hou, L. et al. Optically switchable NIR photoluminescence of PbS semiconducting nanocrystals using diarylethene photoswitches. *J. Am. Chem. Soc.* **144**, 17758–17762 (2022). 10.1021/jacs.2c07102 PMC9545151 36149400

[22] Beharry, A. A. et al. Azobenzene photoswitching without ultraviolet light. *J. Am. Chem. Soc.* **133**, 19684–19687 (2011). 10.1021/ja209239m 22082305

[23] Zhang, Z. W. et al. Stepping out of the blue: from visible to Near-IR triggered photoswitches. *Angew. Chem. Int. Ed.* **61**, e202205758 (2022). 10.1002/anie.202205758 35524420

[24] Satake, K. et al. NIR-responsive double closed-ring isomer of a diarylethene fused dimer synthesized by stepwise photochemical and oxidative cyclization reaction. *J. Am. Chem. Soc.* **147**, 9653–9664 (2025). 10.1021/jacs.4c17757 40053870

[25] Zhang, Z. W. et al. Diarylethenes with a narrow singlet-triplet energy gap sensitizer: a simple strategy for efficient visible-light photochromism. *Adv. Opt. Mater.* **6**, 1700847 (2018).

[26] Boyer, J. C. et al. Two-way photoswitching using one type of near-infrared light, upconverting nanoparticles, and changing only the light intensity. *J. Am. Chem. Soc.* **132**, 15766–15772 (2010). 10.1021/ja107184z 20949969

[27] Wang, W. H. et al. Vintages for new fashion: red-shifted photoswitching via the triplet-photoreaction channel with charge-transfer complex sensitizers. *J. Am. Chem. Soc.* **147**, 5486–5494 (2025). 10.1021/jacs.4c18682 39879537

[28] Zhang, Z. W. et al. Thermo-bistable red and sensitized near-infrared photoswitches. *J. Am. Chem. Soc.* **148**, 6474–6484 (2026). 10.1021/jacs.5c20020 41636160

[29] Li, Z. et al. Correlation of CdS nanocrystal formation with elemental sulfur activation and its implication in synthetic development. *J. Am. Chem. Soc.* **133**, 17248–17256 (2011). 10.1021/ja204538f 21939230

[30] Huang, W. H. et al. Size tunable and controllable synthesis of PbS quantum dots for broadband photoelectric response. *Opt. Mater.* **142**, 113977 (2023).

[31] Chen, K. et al. An efficient all-visible light-activated photoswitch based on diarylethenes and CdS quantum dots. *Chem. Sci.* **15**, 20365–20370 (2024). 10.1039/d4sc06110a PMC11577264 39574538

[32] Irie, M. et al. Photochromism of diarylethene molecules and crystals: memories, switches, and actuators. *Chem. Rev.* **114**, 12174–12277 (2014). 10.1021/cr500249p 25514509

[33] Luo, X. et al. Triplet energy transfer from CsPbBr_3_ nanocrystals enabled by quantum confinement. *J. Am. Chem. Soc.* **141**, 4186–4190 (2019). 10.1021/jacs.8b13180 30817139

[34] Luo, X. et al. Mechanisms of triplet energy transfer across the inorganic nanocrystal/organic molecule interface. *Nat. Commun.* **11**, 28 (2020). 10.1038/s41467-019-13951-3 PMC6946700 31911606

[35] Liu, M. et al. Coherent manipulation of photochemical spin-triplet formation in quantum dot-molecule hybrids. *Nat. Mater.* **24**, 260–267 (2025). 10.1038/s41563-024-02061-1 39762496

[36] Cnossen, A. et al. Driving unidirectional molecular rotary motors with visible light by intra- and intermolecular energy transfer from palladium porphyrin. *J. Am. Chem. Soc.* **134**, 17613–17619 (2012). 10.1021/ja306986g 23036108

[37] Yano, N. et al. Photoluminescence on/off switching of a single colloidal quantum dot using photochromic diarylethene. *J. Phys. Chem. C.* **124**, 17423–17429 (2020).

[38] Chen, K. et al. Optical modulation of photoluminescence in carbon quantum dots using diarylethene molecular photoswitches [Invited]. *Chin. Opt., Lett.* **23**, 021601 (2025).

[39] Hou, L. et al. A general approach for all-visible-light switching of diarylethenes through triplet sensitization using semiconducting nanocrystals. *J. Mater. Chem. C.* **10**, 15833–15842 (2022).

[40] Yu, W. W. et al. Experimental determination of the extinction coefficient of CdTe, CdSe, and CdS nanocrystals. *Chem. Mater.* **15**, 2854–2860 (2003).

[41] Moreels, I. et al. Size-dependent optical properties of colloidal PbS quantum dots. *ACS Nano* **3**, 3023–3030 (2009). 10.1021/nn900863a 19780530

