# Supplementary material for: Switchable quantum dot photoluminescence across a broad spectrum with all-visible-light activated diarylethene photoswitches
Source: Nat Commun. 2026 Oct 2;17:11285. doi: 10.1038/s41467-026-78224-2 (PMC13720203; doi:10.1038/s41467-026-78224-2)
Supplement: Supplementary file 1 [file 41467_2026_78224_MOESM1_ESM.pdf]

Switchable quantum dot photoluminescence across a broad spectrum with all-visible-light activated diarylethene photoswitches

Lin Xi[1], Kezhou Chen[1], Shuai Zhang[1]*, Taibin Wang[1], Tiegen Liu[1], Bo Albinsson[2]*, Lili Hou[1,2]*

1. State Key Laboratory of Precision Measurement Technology and Instruments, Key Laboratory of Opto-electronics Information Technology, School of Precision Instrument and Opto-Electronics Engineering, Tianjin University, Tianjin 300072, China
2. Department of Chemistry and Chemical Engineering, Chalmers University of Technology, Gothenburg 412 96, Sweden

E-mail: lilihou@tju.edu.cn; balb@chalmers.se; shuaizhang90@tju.edu.cn

1 Characterization of CdS and PbS QDs

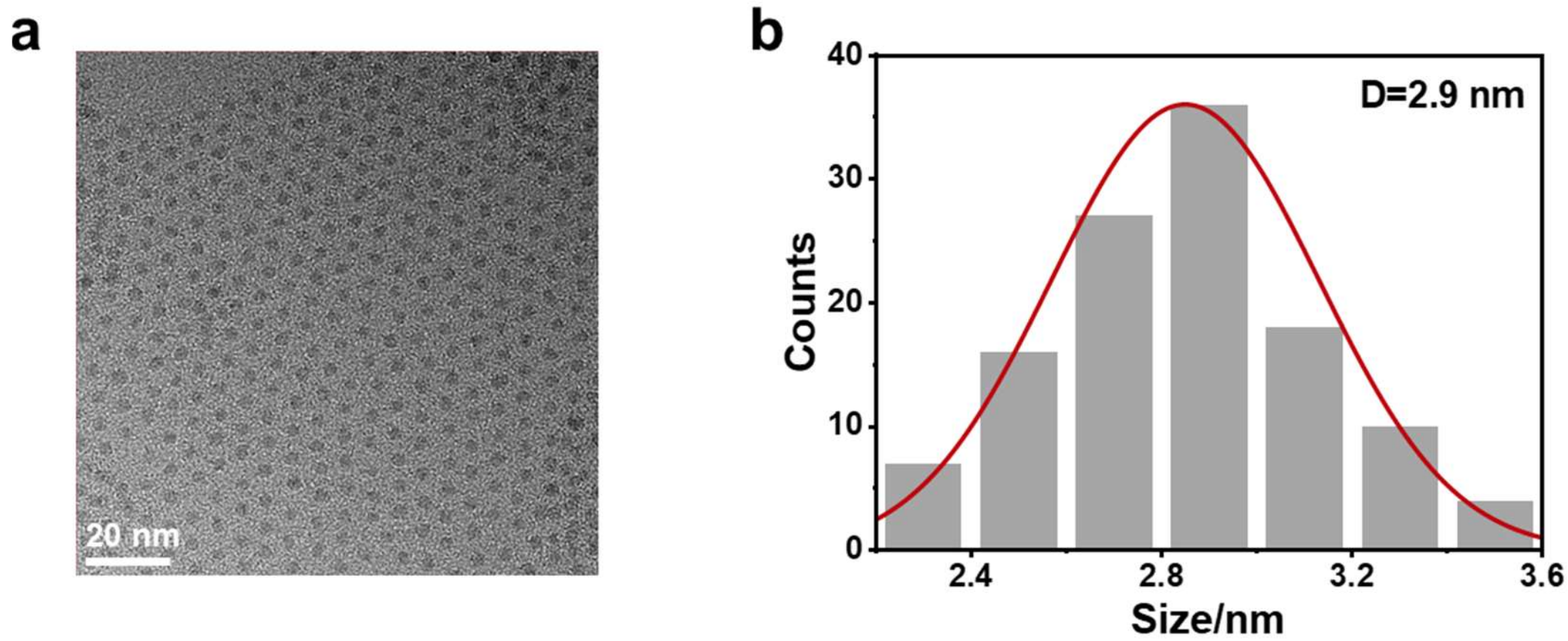

Supplementary Fig. 1 Morphology of CdS QDs. a TEM image of CdS QDs. **b** Size distribution histogram of CdS QDs. Source data are provided as a Source Data file.

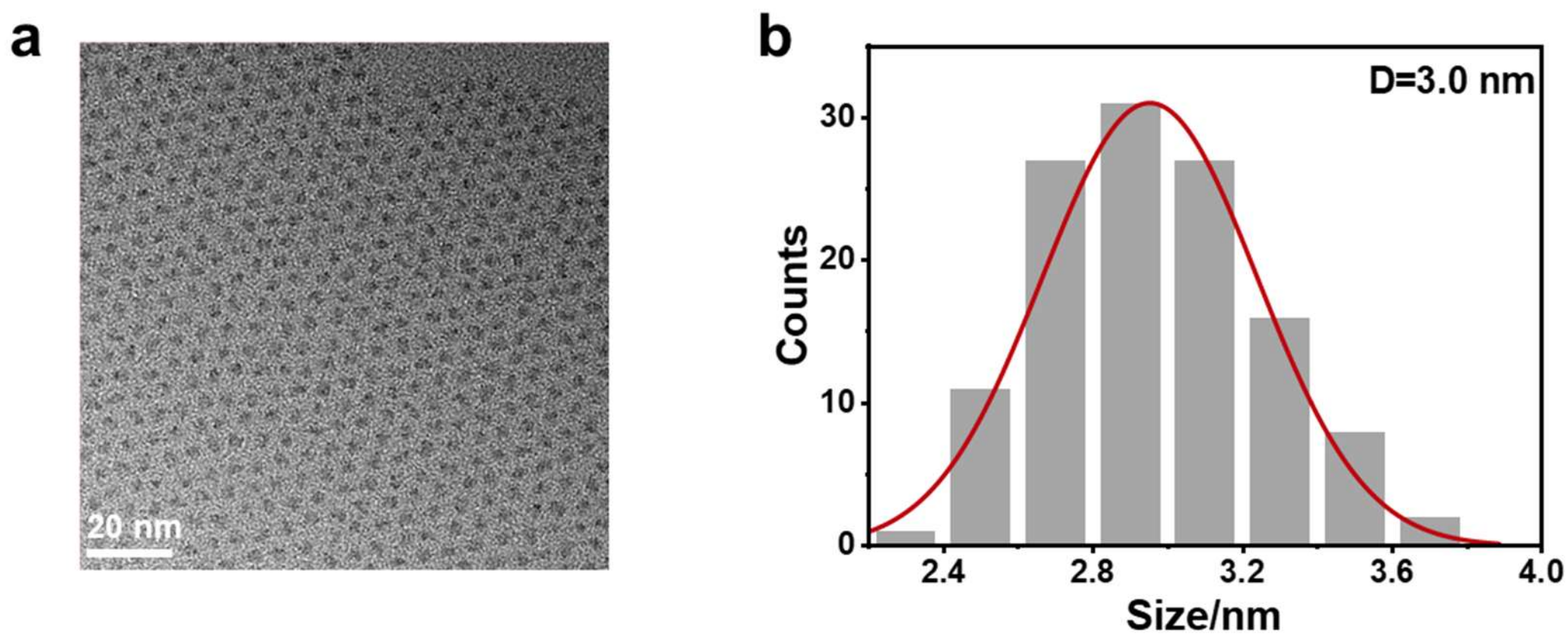

Supplementary Fig. 2 Morphology of PbS QDs. a TEM image of PbS QDs. **b** Size distribution histogram of PbS QDs. Source data are provided as a Source Data file.

2 Femtosecond transient absorption spectra of QDs with and without DAE

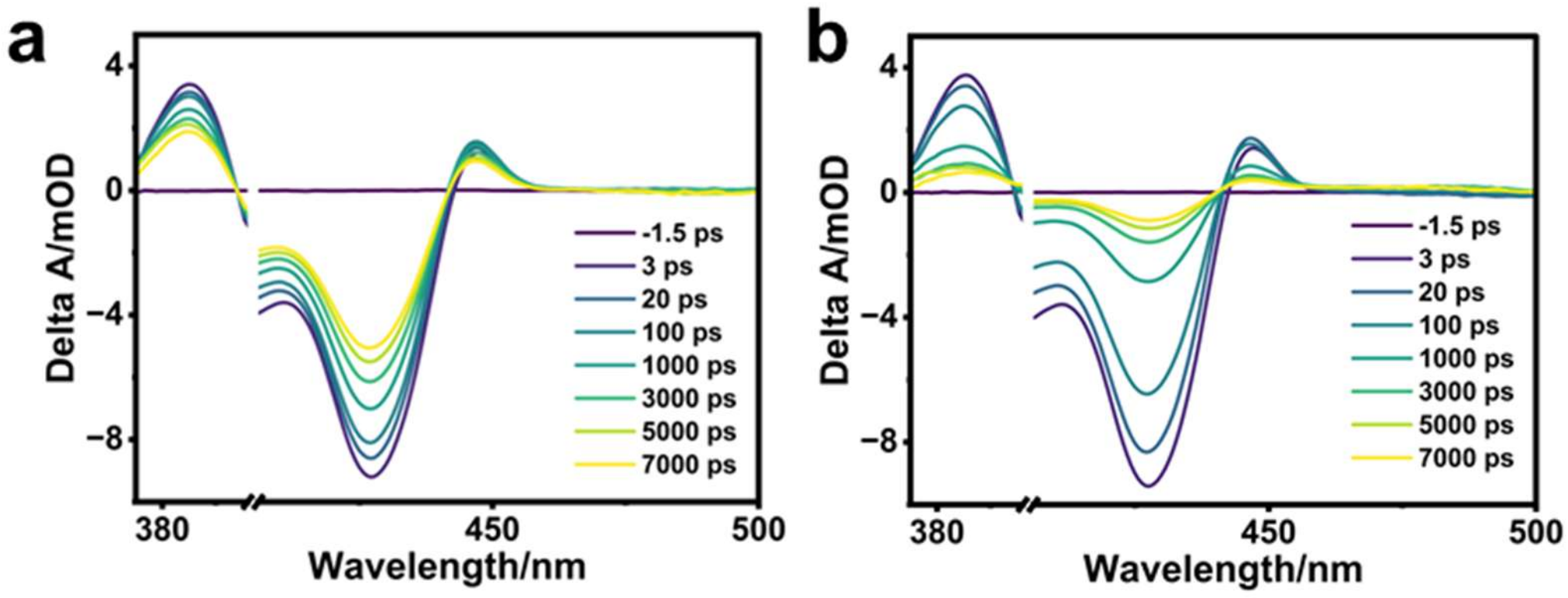

Supplementary Fig. 3 Femtosecond transient absorption (fsTA) spectra of CdS QDs without and with DAE. a fsTA spectra of CdS QDs. **b** fsTA spectra of CdS QD/o-DAE mixture. All the samples were excited at 400 nm. Source data are provided as a Source Data file.

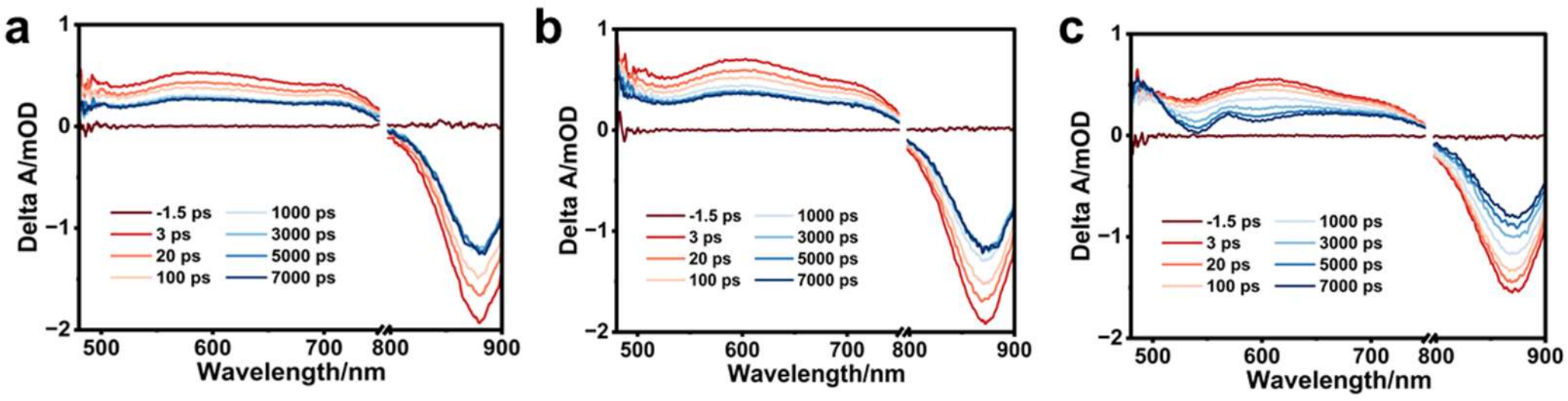

Supplementary Fig. 4 fsTA spectra of PbS QDs without and with c-DAE and o-DAE. a fsTA spectra of PbS QD. **b** fsTA spectra of PbS QD/o-DAE mixture. **c** fsTA spectra of PbS QD/c-DAE mixture. All the samples were excited at 780 nm. Source data are provided as a Source Data file.

3 Optical characterizations of the PbS QD/non-carboxylated DAE/CdS QD mixture

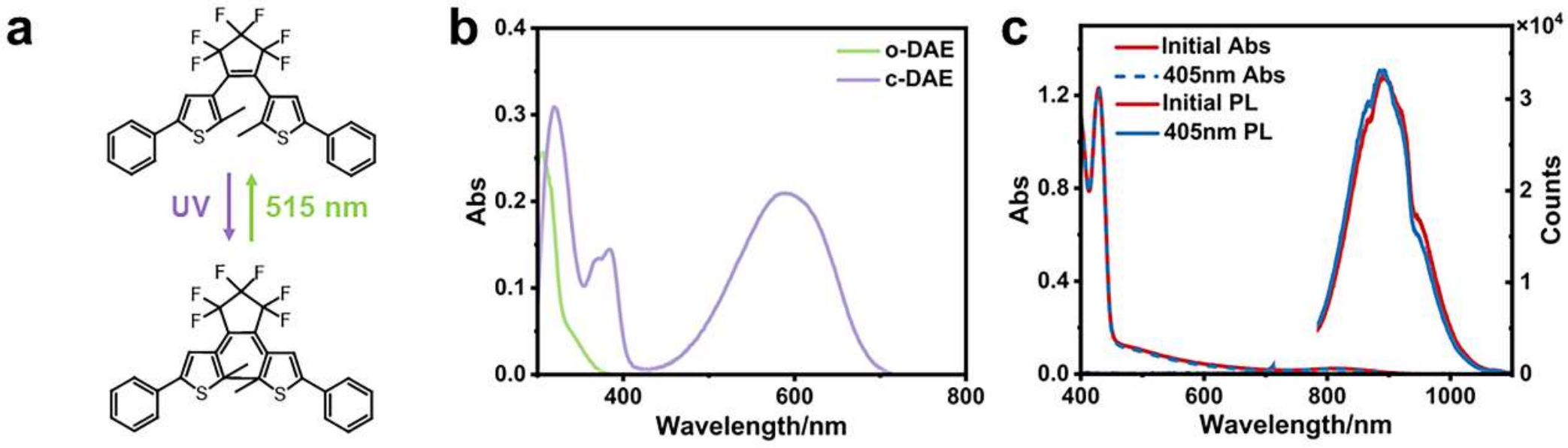

Supplementary Fig. 5 Characterization of the non-carboxylated DAE system. a Chemical structure of non-carboxylated DAE in the open and closed forms. **b** Absorption spectra of non-carboxylated DAE in the open and closed forms. **c** Absorption and PL spectra of PbS QD/non-carboxylated DAE/CdS QD mixture in the initial state and after 405 nm irradiation. The emission signal at <760 nm is removed due to scattering of the excitation light. All PL spectra were recorded under 760 nm excitation. Source data are provided as a Source Data file.

4 Control experiment of the PbS QD/DAE mixture with and without CdS QDs

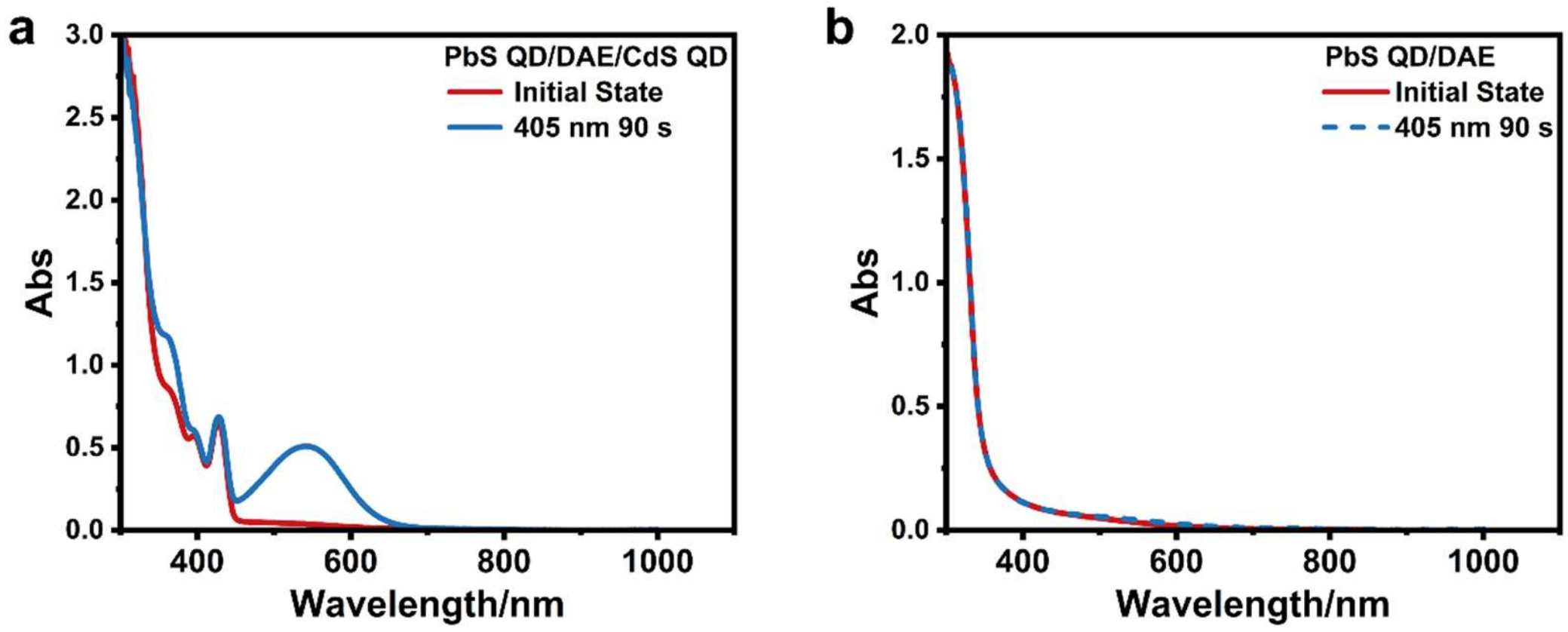

Supplementary Fig. 6 UV-vis absorption spectra of QD/DAE mixture under 405 nm light irradiation. UV-vis absorption spectra of the PbS QD/DAE mixture **a** with and **b** without CdS QDs under 405 nm irradiation for 90 s. Source data are provided as a Source Data file.

5 PL behavior of PbS QDs under alternating 405 nm and 515 nm irradiation

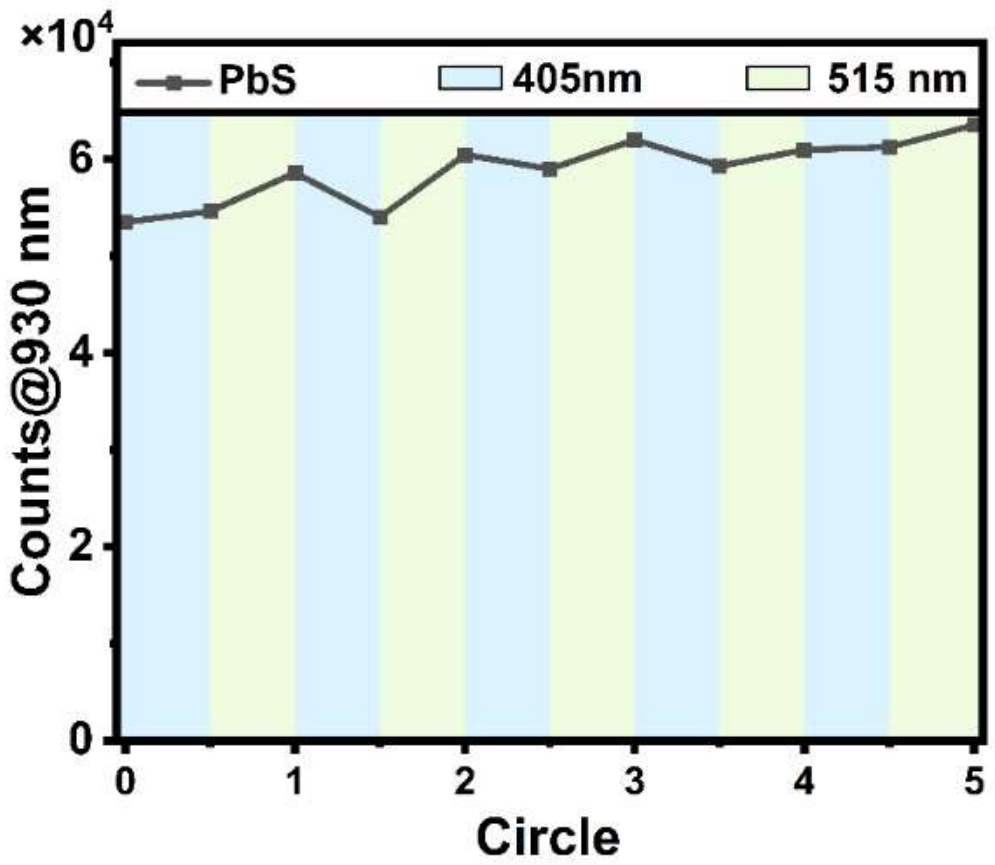

Supplementary Fig. 7 PL behavior of bare PbS QDs. PL intensity of bare PbS QDs under alternating 405 nm and 515 nm irradiation (excited at 760 nm, monitored at 930 nm). Source data are provided as a Source Data file.

6 Lifetime of PbS QDs with c-DAEs and CdS QDs at a molar concentration ratio of 1:80:4

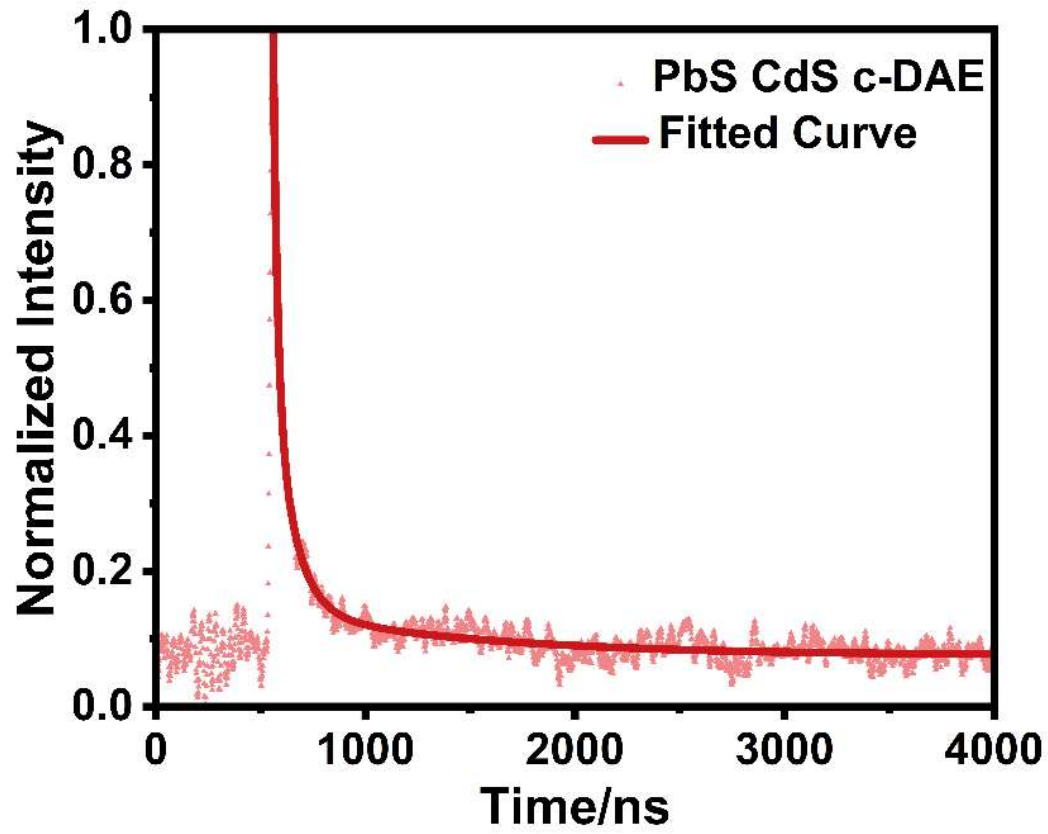

Supplementary Fig. 8 Time-resolved PL decay of the PbS QD/c-DAE/CdS QD system. PL lifetime of PbS QDs with c-DAE and CdS QDs at a molar ratio of 1:80:4 in toluene. The sample was excited at 700 nm and the lifetime was monitored at 900 nm. The solid line indicates fits of three exponentials decay. Source data are provided as a Source Data file.

The time-resolved PL PbS QDs exhibit complex multi exponential decays as reported.[1] Herein three exponentials decay was used to achieve a satisfactory fit:[2]

$$I = I_0 \sum_{i=1}^{3} A_i e^{-t/\tau_i} \tag{1}$$

where A_i and τ_i are the amplitude and lifetime of the *i*:th exponential component, respectively. The amplitude-weighted average lifetimes were calculated using equation 6

$$\langle \tau \rangle_{avg} = \frac{\sum_{i=1}^{3} A_i \tau_i}{\sum_{i=1}^{3} A_i} \tag{2}$$

Supplementary Table 1. PL lifetimes of PbS QDs with different c-DAE concentrations.

c-DAE (μM)	A_1	τ_1 (ns)	A_2	τ_2 (ns)	A_3	τ_3 (ns)	$<\tau>_{avg}$ (ns)
0	0.02	130.8	0.12	4205.0	0.69	1808.7	2114.6
2.5	0.31	11. 4	0.21	312.8	0.49	2131.1	1100.4
5	0.44	19.3	0.33	287.3	0.34	1987.2	698.1
7.5	0.18	36.2	0.32	298.1	0.32	1982.8	893.1
10	0.21	25.7	0.36	240.5	0.24	1745.5	620.8
15	0.22	42.5	0.38	250.7	0.18	1650.5	515.7

Supplementary Table 2 PL lifetime of PbS QDs with concentration ratio of PbS QDs to c-DAE of 1:80.

Sample	A_1	τ_1 (ns)	A_2	τ_2 (ns)	A_3	τ_3 (ns)	$<\tau>_{avg}$ (ns)
PbS/ c-DAE/ CdS	0.28	92.1	0.32	19.4	0.05	975.3	122.3

7 Characterization of blue, green, red and white color QDs

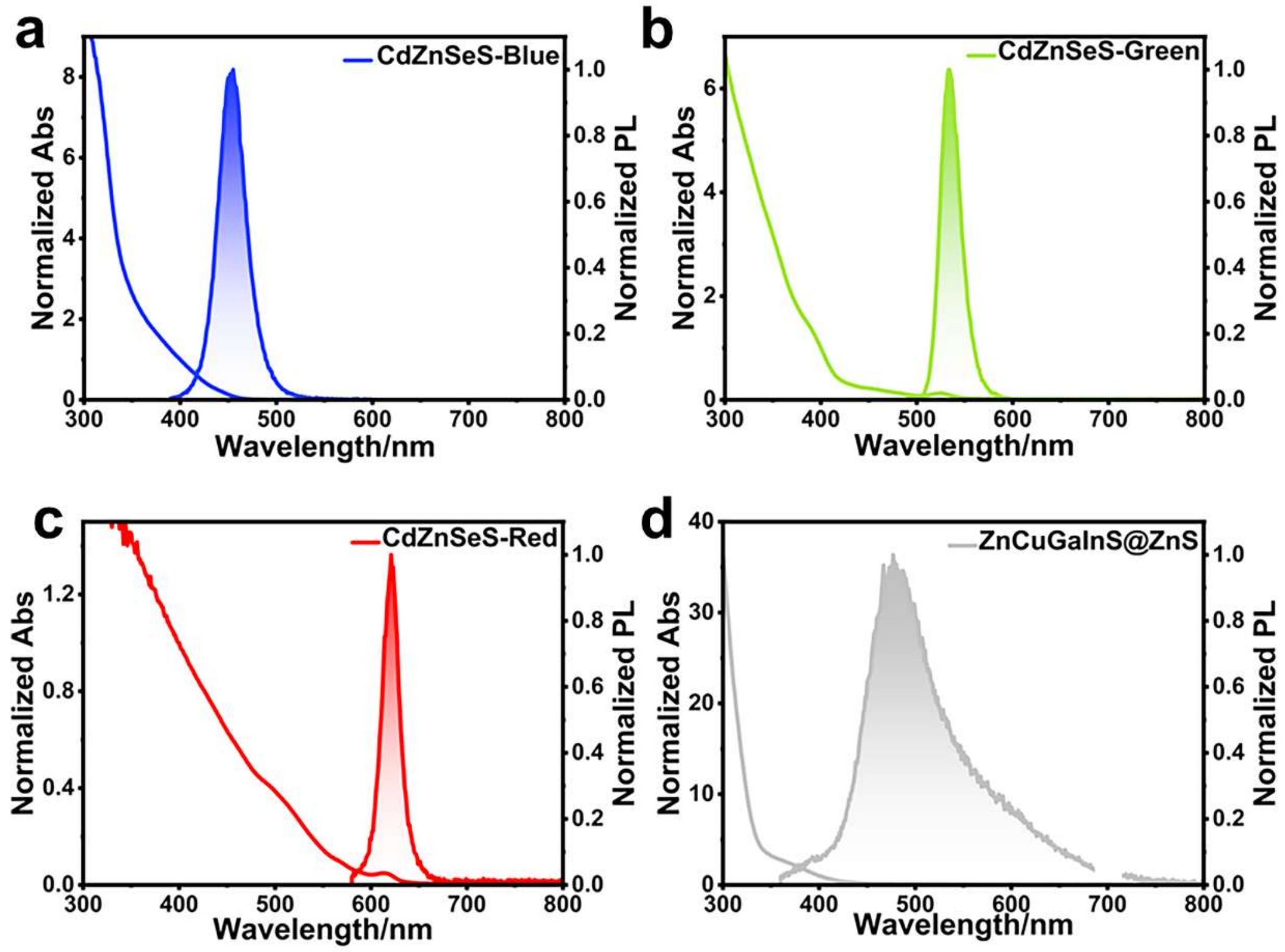

Supplementary Fig. 9 Optical characterization of PL QDs. The absorption and PL spectra of CdZnSeS gradient alloy QDs with **a** blue, **b** green, and **c** red emission, and **d** ZnCuGaInS@ZnS QDs exhibiting broad emission covering the whole visible range and appears to emit white light. The emission signal at ~700 nm in **d** is removed due to the second order scattering of the excitation light. Spectra are normalized to the absorption at 400 nm and to the maximum of the respective PL spectra. Source data are provided as a Source Data file.

8 Absorption spectra of blue, green, red and white color QDs with DAE and CdS QDs

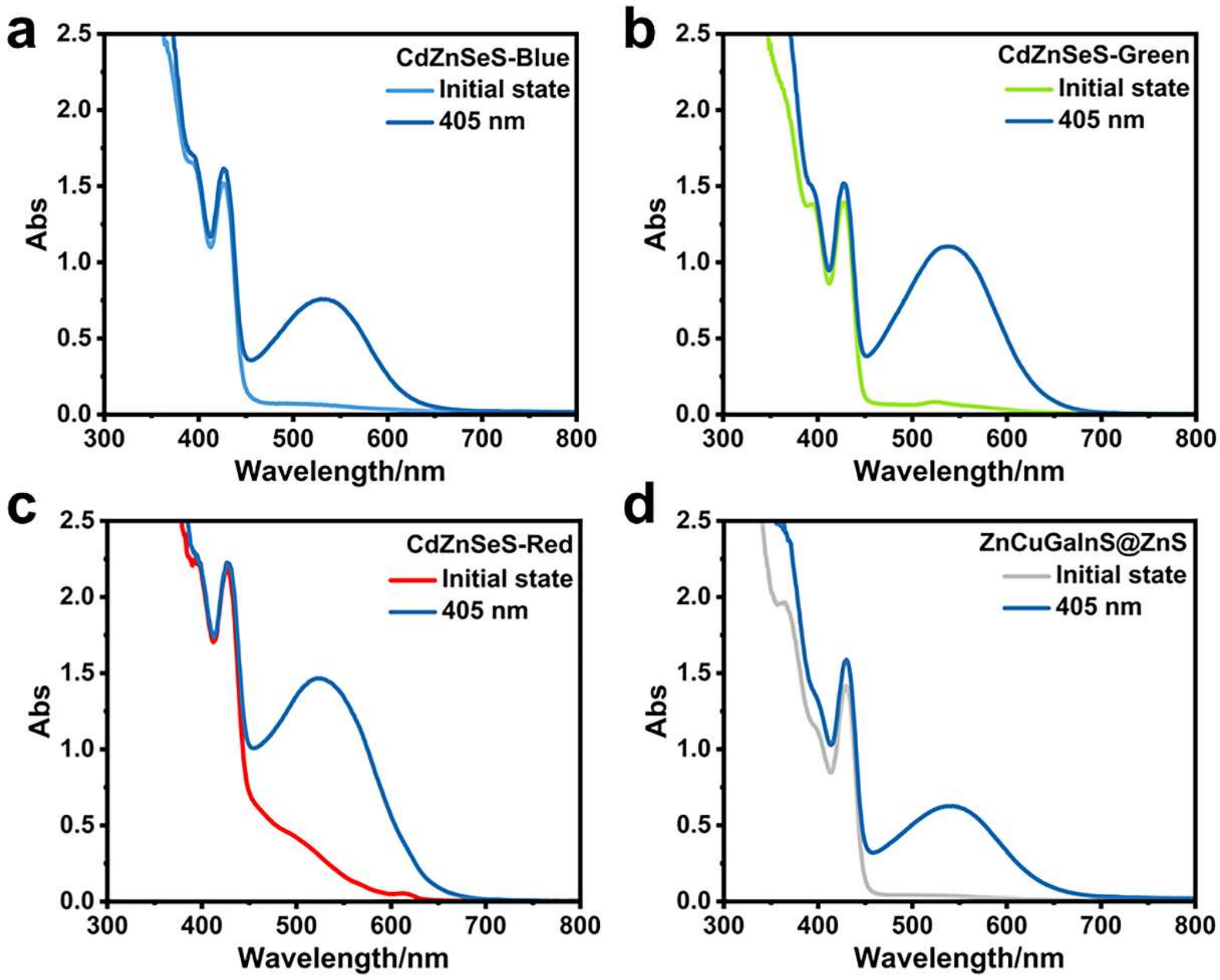

Supplementary Fig. 10 Absorption spectra of QD/DAE/CdS PL QD mixtures. The absorption spectra of mixtures in the initial state and photostationary state under 405 nm irradiation, in which the PL QD is CdZnSeS gradient alloy QDs with **a** blue, **b** green, and **c** red emission, and **d** ZnCuGaInS@ZnS QDs exhibiting broad emission covering the whole visible range and appears to emit white light. Source data are provided as a Source Data file.

Supplementary Table 3. Comparison of photoswitchable QD combined with photochromic molecules reported in this work and previous studies.

System	Spectral range (nm)	Switch-off*	Switch-on*	On/off ratio	Fatigue resistance	Ref
CdZnSeS gradient alloy QDs /DAE/ CdS QDs	390-530	405 nm, 90 s, 19.1 mW/cm^2	515 nm, 3 min, 198 mW/cm^2	14.3	83.3% after 5 cycles	This work
	500-600			250	90.2% after 5 cycles	
	580-670			20.4	~100% after 5 cycles	
PbS QDs /DAE/ CdS QDs	800-1100			15.6	69.3% after 5 cycles	
PbS QD/DAE	700-900	302 nm, 60 s, 10 mW/cm^2	523 nm, 60 s, 15 mW/cm^2	>100	4 cycles	2022[3]
$CsPbBr_3$ QD/DAE	480-550	365 nm, 6 min, 1.7 mW/cm^2	400-750 nm, 10 min, 13 mW/cm^2	>100	3 cycles	2022[4]
CdSe QD/ FFC	525-675	365 nm, 27 min, 0.6 mW	532 nm, -, 1.6 mW	~5	6 cycles	2021[5]
CdSe/ZnS QD/DAE	500-600	300 nm, 10 s, 100 μW/cm^2	630 nm, 60 s, 150 mW/cm^2	~10	-	2020[6]
CdTe QD/Azo	450-550	300 nm, -	Stored in dark, -	~16	4 cycles	2019[7]
$CsPbX_3$ QD/DAE	420-500	365 nm, 5 min, 0.5 mW	>530 nm, 5 min, 150 mW	~2	3 cycles	2019[8]
	480-560			~10		
	530-700			~10		
CdSe/CdS/ZnS QD/DAE	500-625	340 nm, 100 s, 1 mW/cm^2	550 nm, 40 s, 8.2 mW/cm^2	~1.67	-	2015[9]
CdSe/ZnS QD/DAE	500-600	340 nm, 60 s, 1 mW/cm^2	545 nm, 60 s, 5.8 mW/cm^2	~1.5	16 cycles	2011[10]
CdSe/ZnS QD/SP	440-560	350 nm, 5 s, 0.97 mW	588 nm, 90 s, 20 mW	~8	4 cycles	2005[11]

* Switch off/on illumination parameters consist of incident light wavelength, irradiation time and optical power density.

9 Stability of the multi-level data storage sample in the dark

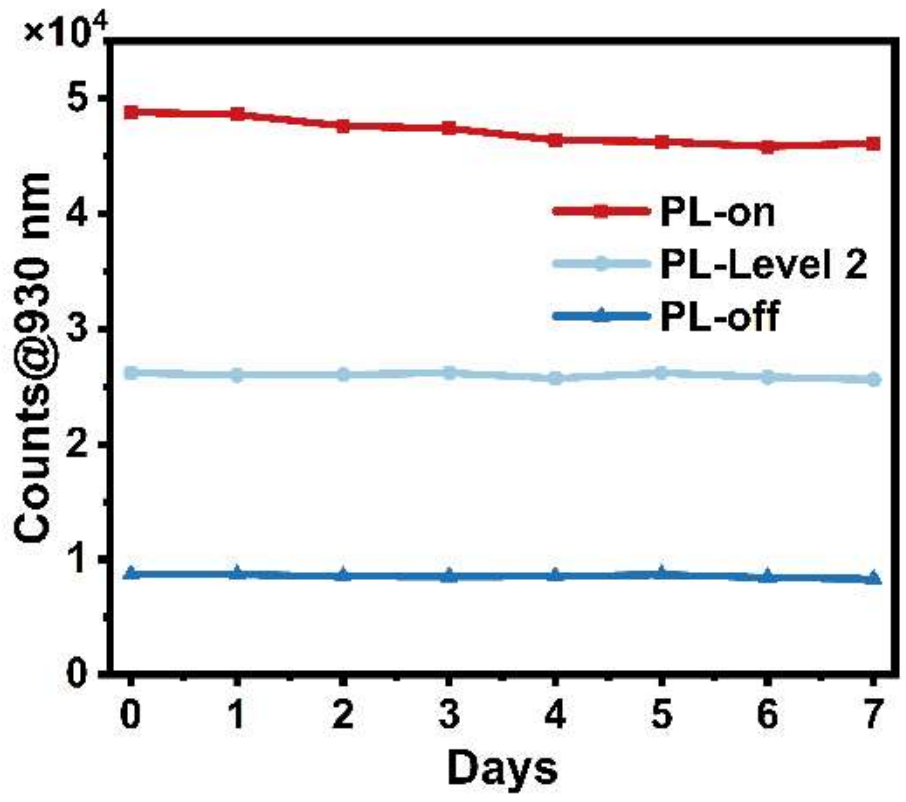

Supplementary Fig. 11 Stability of three storage states for NIR emission samples. PL intensity variation of on, level-2, and off states of the PbS QD/DAE/CdS QD sample over 7 days. All samples were stored in the dark under ambient atmosphere at 24 °C and 25% relative humidity. Source data are provided as a Source Data file.

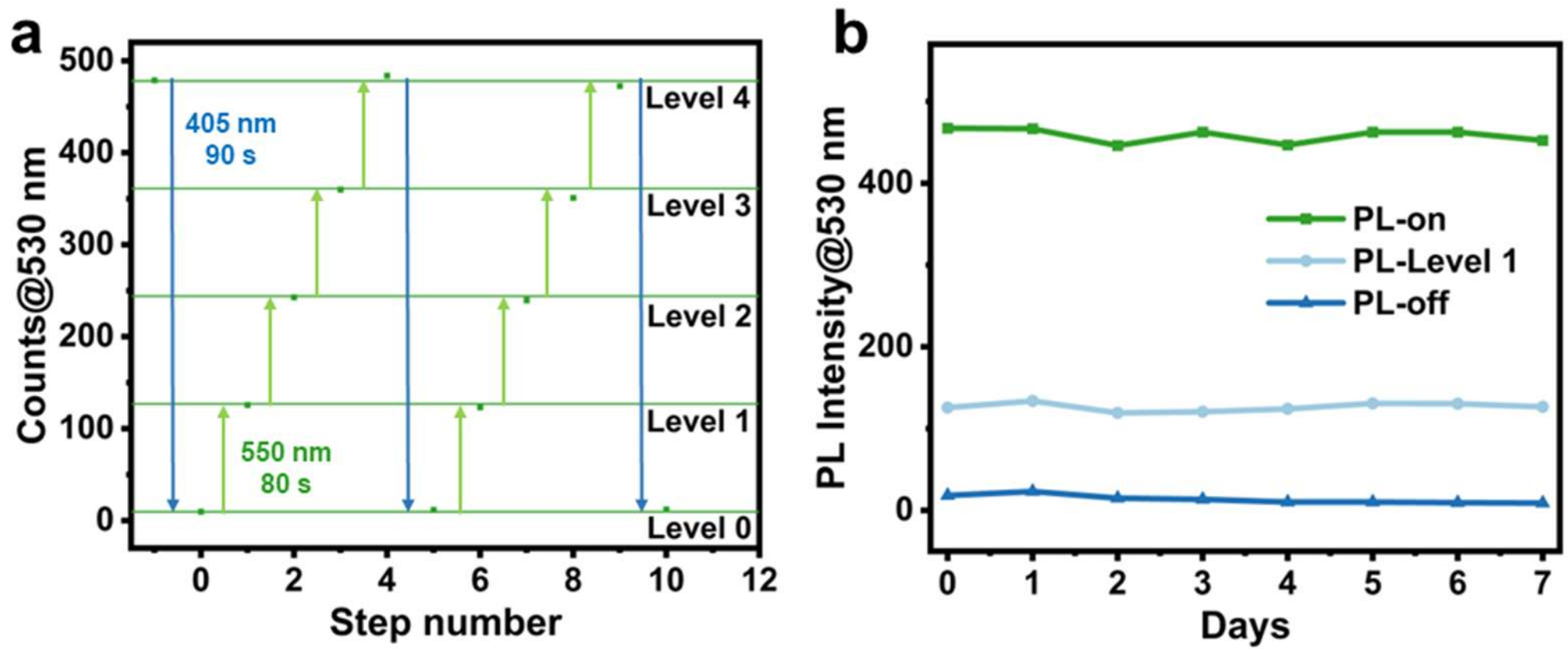

Supplementary Fig. 12 Stability of different storage states in GQD/DAE/CdS QD solution samples. a The signal at 530 nm was divided into 4 levels by an interval of 117 a.u. (defined as a single quantization level). The corresponding writing time for each level was set to 80 s under 550 nm light irradiation. b PL intensity variation of on, level-1, and off states of the GQD/DAE/CdS QD solution sample over 7 days. Source data are provided as a Source Data file.

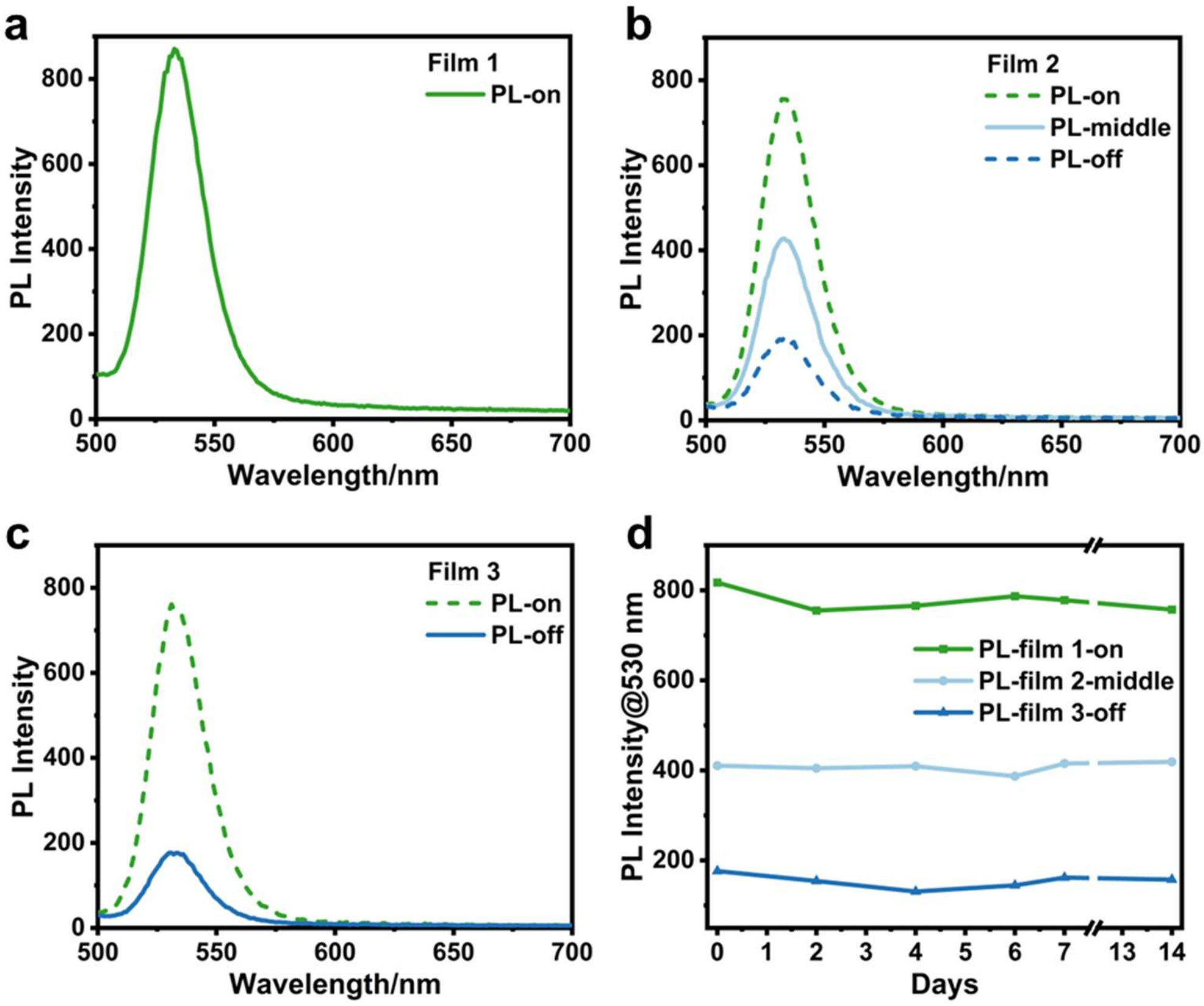

Supplementary Fig. 13 Stability of different storage states in GQD/DAE/CdS QD thin films. a-c PL spectra of three thin films representing the calibrated on, middle, and off states. **d** PL intensity variation of the corresponding storage states during storage for 14 days in the dark under ambient atmosphere at 24 °C and 25% relative humidity. The PL intensity of each state was referenced to the corresponding calibrated film. Source data are provided as a Source Data file.

Supplementary references

1. Greben, M. *et al.* Deciphering the emission dynamics of colloidal PbS quantum dots: a spectroscopic exploration under diverse environmental and experimental conditions. *Nanoscale* **17**, 1602-1615 (2025).
2. Hou, L. *et al.* A general approach for all-visible-light switching of diarylethenes through triplet sensitization using semiconducting nanocrystals. *J. Mater. Chem. C* **10**, 15833-15842 (2022).
3. Hou, L. *et al.* Optically switchable NIR photoluminescence of PbS semiconducting nanocrystals using diarylethene photoswitches. *J. Am. Chem. Soc.* **144**, 17758-17762 (2022).
4. Akaishi, Y. *et al.* Light-stimulated luminescence control of lead halide-based perovskite nanocrystals coupled with photochromic molecules via electron and energy transfer. *Small* **18**, 2205046 (2022).
5. Padgaonkar, S. *et al.* Light-triggered switching of quantum dot photoluminescence through excited-state electron transfer to surface-bound photochromic molecules. *Nano Lett.* **21**, 854-860 (2021).
6. Yano, N. *et al.* Photoluminescence on/off switching of a single colloidal quantum dot using

photochromic diarylethene. *J. Phys. Chem. C* **124**, 17423-17429 (2020).
7. Saeed, S. *et al.* Photoresponsive azobenzene ligand as an efficient electron acceptor for luminous CdTe quantum dots. *J. Photoch. Photobio. A* **375**, 48-53 (2019).
8. Akaishi, Y. *et al.* Reversible ON/OFF switching of photoluminescence from $CsPbX_3$ quantum dots coated with silica using photochromic diarylethene. *Chem. Commun.* **55**, 8060-8063 (2019).
9. Díaz, S.A. *et al.* Photoswitchable semiconductor nanocrystals with self-regulating photochromic Forster resonance energy transfer acceptors. *Nat. Commun.* **6**, 6036 (2015).
10. Díaz, S.A. *et al.* Photoswitchable water-soluble quantum dots: pcFRET based on amphiphilic photochromic polymer coating. *ACS Nano* **5**, 2795-2805 (2011).
11. Zhu, L.Y. *et al.* Light-controlled molecular switches modulate nanocrystal fluorescence. *J. Am. Chem. Soc.* **127**, 8968-8970 (2005).
