# Supplementary material for: Switchable quantum dot photoluminescence across a broad spectrum with all-visible-light activated diarylethene photoswitches
Source: Nat Commun. 2026 Oct 2;17:11285. doi: 10.1038/s41467-026-78224-2 (PMC13720203; doi:10.1038/s41467-026-78224-2)
Supplement: Supplementary file 2 [file 41467_2026_78224_MOESM2_ESM.pdf]

nature portfolio

# Peer Review File

# Switchable Quantum Dot Photoluminescence Across a Broad Spectrum with All-visible-light Activated Diarylethene Photoswitches

Corresponding Author: Professor Bo Albinsson

**This file contains all reviewer reports in order by version, followed by all author rebuttals in order by version.**

Version 0:

Reviewer comments:

Reviewer #1

(Remarks to the Author)
This manuscript presents a system for reversible photoluminescence switching of quantum dots using all-visible-light-activated diarylethene photoswitches. The work demonstrates modulation across a broad spectral range (454-950 nm) with impressive quenching efficiencies (up to 99.6%) and on/off ratios (up to 250). Applications in multi-level data storage and anti-counterfeiting are also shown. Overall, this is a technically sound study that further develops the authors' previous work on visible-light-activated photoswitching (e.g., refs 22, 31, 36) to a broader range of quantum dot emitters. The quenching efficiencies are impressive, and the demonstrations in data storage and anti-counterfeiting are promising. I recommend minor revision to allow the authors to address the points below and further strengthen the manuscript.
1. The triplet energy transfer mechanism is invoked but not directly probed. Transient absorption spectroscopy or additional control experiments could strengthen the mechanistic claims.
2. For real-world applications, the long-term stability of the solid-state samples under ambient conditions (oxygen, moisture) would be worth addressing. The current stability data (Fig. S8) is limited to 3 days in the dark. We suggest the authors perform the stability test for at least one week.
3. A table comparing this system's performance (spectral range, switching speeds, on/off ratios, fatigue resistance) with previous UV-activated and visible-light-activated systems would help readers appreciate the advancements.
4. Some sections could benefit from tighter writing. For example, the introduction is somewhat lengthy and could be streamlined to focus more directly on the gap this work addresses.
5. Several latest researches on long-wavelength photoswitch design should be cited, e.g., JACS 2025, 5486; JACS, 2026, 6474.

Reviewer #2

(Remarks to the Author)
This manuscript reports an interesting and efficient strategy that utilizes quantum dots (QDs) and photochromic molecular switches (Diarylethenes, DAEs) to reversibly switch photoluminescence on and off across a broad spectral range under visible light excitation. The authors also demonstrate their strategy for multi-level data storage and anti-counterfeiting in the NIR region, which holds promise for future smart light-responsive applications. Overall, this work represents a successful and excellent example of combining the fields of photoswitches and QDs, and should be of broad interest to researchers in relevant areas. The paper can be published in Nature Communications after addressing a few issues below:

1） A control experiment containing only DAE and PbS QDs (or other QDs with strong photoluminescence) without CdS QDs should be performed. Importantly, the UV-visible absorption spectra should be monitored under purple light irradiation to clarify the role of CdS QDs as triplet sensitizers in achieving all-visible-light functions.
2） The x-scale titles appear to be cut off in Figure 2d-e, and error bars in Figure 2f should be provided and discussed.
3） Since triplet energy transfer (TET) is the key mechanism for achieving both visible activation of DAEs and photoswitching of QD photoluminescence in this work, relevant mechanistic studies in the field of QD triplet sensitization should be appropriately cited and discussed, such as J. Am. Chem. Soc. 2019, 141, 4186–4190; Nat. Commun. 2020, 11, 28; Nat. Mater. 2025, 24, 260–267.

4 ) The authors should also discuss strategies to replace heavy metal-containing QDs to advance photoswitchable photoluminescence toward biocompatible applications.

Version 1:

Reviewer comments:

Reviewer #1

(Remarks to the Author)
The authors have fully addressed the issues raised by all reviewers. The manuscript is much improved in current form and we are happy to read the revised version and to see the efforts the authors made. We consider the present version could meet the quality to be accepted by Nature Communications. Thank you!

Reviewer #2

(Remarks to the Author)
The authors have carefully conducted additional experiments (including transient spectroscopy, stability and control measurements) to address reviewers' comments. The revised manuscript is suitable for publication.

**Open Access** This Peer Review File is licensed under a Creative Commons Attribution 4.0 International License, which permits use, sharing, adaptation, distribution and reproduction in any medium or format, as long as you give appropriate credit to the original author(s) and the source, provide a link to the Creative Commons license, and indicate if changes were made.
In cases where reviewers are anonymous, credit should be given to 'Anonymous Referee' and the source.
The images or other third party material in this Peer Review File are included in the article's Creative Commons license, unless indicated otherwise in a credit line to the material. If material is not included in the article's Creative Commons license and your intended use is not permitted by statutory regulation or exceeds the permitted use, you will need to obtain permission directly from the copyright holder.
To view a copy of this license, visit https://creativecommons.org/licenses/by/4.0/

# Point-by-point Reply

Dear Reviewers,

Thank you for taking time to carefully review our manuscript. We appreciate your thoughtful feedback and very constructive comments. The revised manuscript has addressed all comments raised, and the changes in the main text and supplementary information (SI) are highlighted in yellow. Additional experiments, including transient spectroscopy, stability and control measurements, have been carried out and the results have been interpreted in response to the comments of the reviewers. As suggested by one of the Reviewers, we have also compared the present work with previous publications and summarized the results in a table. Please find below our detailed point-by-point reply, as well as the corresponding modifications to the main text and SI.

Thanks again for reviewing our manuscript and insightful comments.

On behalf of all co-authors:

Sincerely yours,

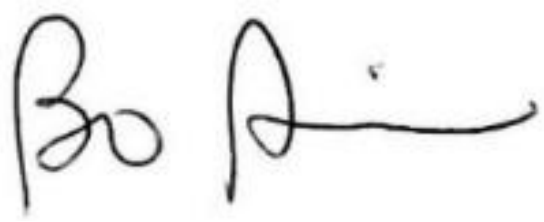

***Dr. Bo Albinsson***
***Professor in Physical Chemistry***

CHALMERS UNIVERSITY OF TECHNOLOGY
Department of Chemistry and Chemical Engineering
Division of Chemistry and Biochemistry
SE-412 96 Gothenburg, SWEDEN
Phone: 46-31-772 3044
Email: balb@chalmers.se

**Reviewer: 1**

*Comments:*

*This manuscript presents a system for reversible photoluminescence switching of quantum dots using all-visible-light-activated diarylethene photoswitches. The work demonstrates modulation across a broad spectral range (454-950 nm) with impressive quenching efficiencies (up to 99.6%) and on/off ratios (up to 250). Applications in multi-level data storage and anti-counterfeiting are also shown. Overall, this is a technically sound study that further develops the authors' previous work on visible-light-activated photoswitching (e.g., refs 22, 31, 36) to a broader range of quantum dot emitters. The quenching efficiencies are impressive, and the demonstrations in data storage and anti-counterfeiting are promising. I recommend minor revision to allow the authors to address the points below and further strengthen the manuscript.*

**Reply to general comment:**

We appreciate the Reviewer's summary of the key aspects of our manuscript and for recognizing the breakthrough we have achieved in this work. We also thank the Reviewers for the constructive comments, which help us to further improve the quality of this work. In response to these comments, additional stability experiments have been carried out. As suggested, we have included a comparative table summarizing the current work and related studies published previously. To improve clarity and readability, we have also streamlined the introduction part of the manuscript. Detailed point-by-point responses are provided below, and the corresponding changes have been highlighted in the revised manuscript and the SI.

1. *The triplet energy transfer mechanism is invoked but not directly probed. Transient absorption spectroscopy or additional control experiments could strengthen the mechanistic claims.*

**Reply to comment 1.1:**

We thank the Reviewer for raising this important point regarding the mechanistic investigation. To directly probe the proposed triplet energy transfer (TET) mechanism, femtosecond transient absorption (TA) spectroscopy has been performed. The TA spectra, provided as Supplementary Fig. 3 and 4 in the revised SI, show the evidences of efficient triplet energy transfer and the formation of triplet state of DAEs, supporting that the TET process from QDs to c-DAE is the dominant mechanism in our design. In addition, the TA spectra and revealed mechanism is consistent with our previous studies (now cited as Ref 21 and 31), in which the triplet sensitization process has been systematically investigated and discussed.

**Our actions to comment 1.1:**

1. **Supplementary Fig. 3** and **Supplementary Fig. 4** have been added in SI as follow:

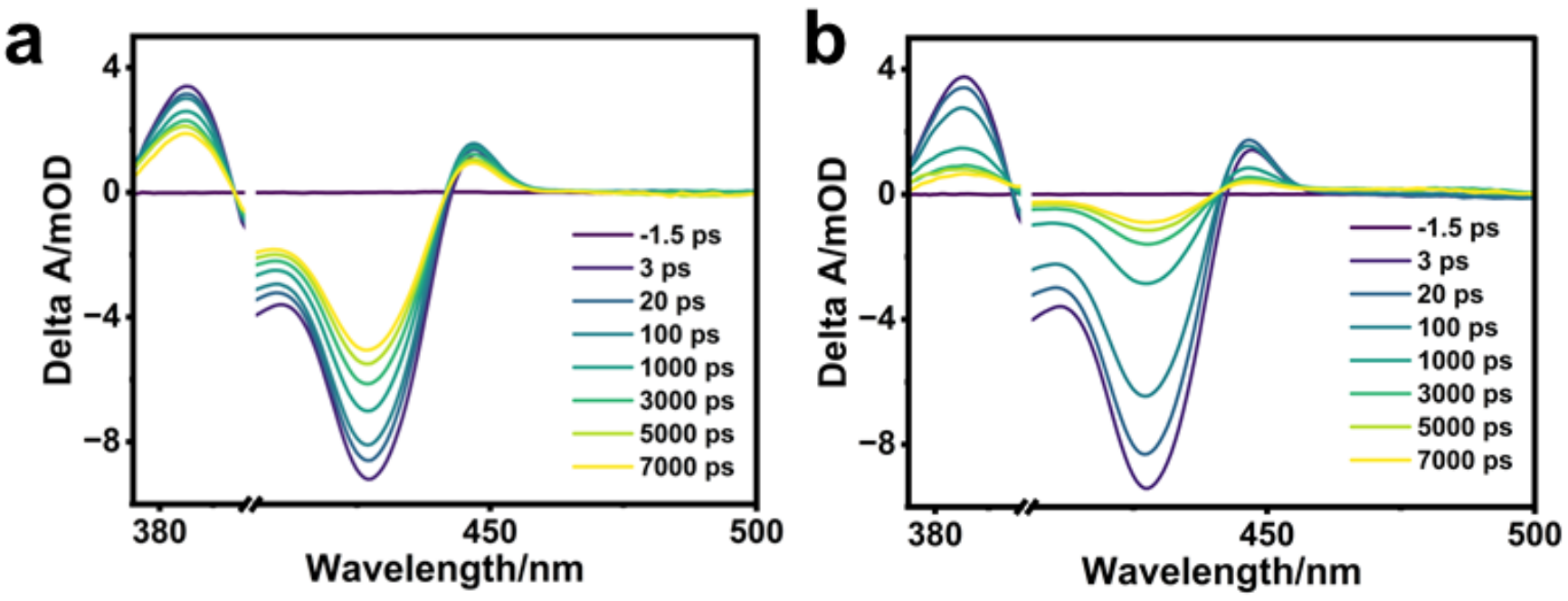


**Supplementary Fig. 3 Femtosecond transient absorption (fsTA) spectra of CdS QDs without and with DAE. a** fsTA spectra of CdS QDs. **b** fsTA spectra of CdS QD/o-DAE mixture. All the samples were excited at 400 nm.

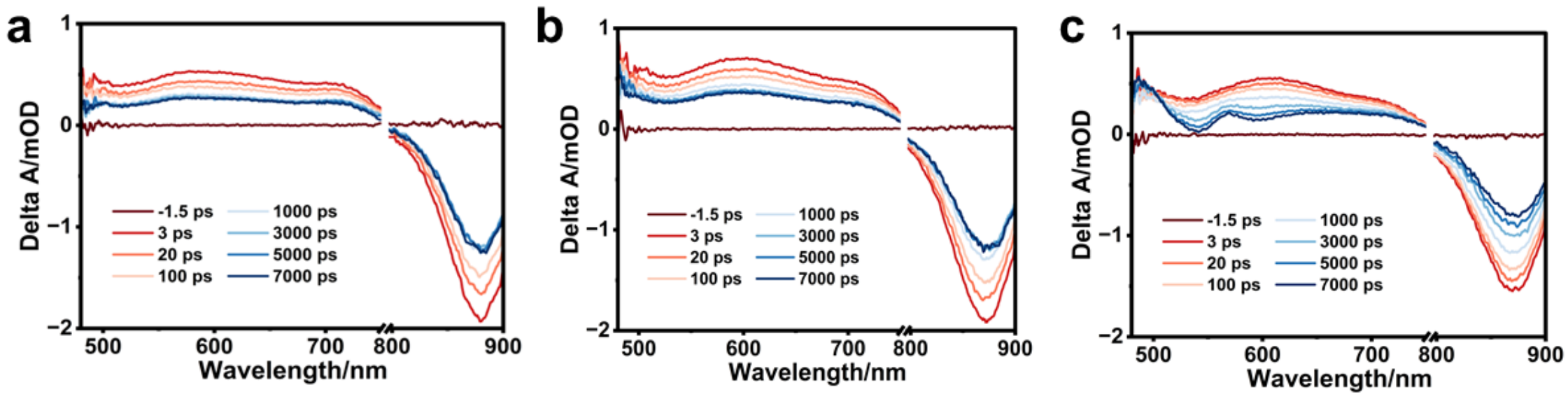


**Supplementary Fig. 4 fsTA spectra of PbS QDs without and with c-DAE and o-DAE. a** fsTA spectra of PbS QDs. **b** fsTA spectra of PbS QD/o-DAE mixture. **c** fsTA spectra of PbS QD/c-DAE mixture. All the samples were excited at 780 nm.

2. In the middle of Page 8 in the revised main text, the discussion of fsTA spectra and the mechanism have been added as follow:

The key to facilitating efficient all-visible-light controlled photoswitching in our design is through two-step TET from CdS QDs and PbS QDs to DAEs. To evidence the TET processes from QDs to DAE, femtosecond transient absorption spectroscopy (fsTA) was performed and the spectra are shown in Supplementary Fig. 3 and 4. The CdS QDs display an excited-state absorption (ESA) around 385 nm and a ground-state bleach (GSB) around 430 nm. After introducing o-DAE, the transient signal of CdS QDs changes notably faster. Specifically, the GSB signal recovered to 55.0% of its initial amplitude at 7 ns for bare CdS QDs, whereas only 9.5% of the initial bleach remained in the CdS QD/o-DAE mixture. No new spectral features are observed within the time window of our pump-probe delay stage (7 ns), suggesting the absence of significant electron transfer. FRET from CdS QDs to DAE can also be ruled out, as there is no overlap between the absorption spectra of o-DAE and the PL spectrum of CdS QDs. The TET process between PbS QDs and DAE was also probed. Compared with bare PbS QDs and the PbS QD/o-DAE mixture, the PbS QD/c-DAE mixture exhibits accelerated recovery of GSB signals of PbS QDs around 875 nm. At 7 ns, the GSB signal retained 64.8% and 63.2% of its initial amplitude for bare PbS QDs and the PbS QD/o-DAE mixture, respectively, whereas only 52.0% remained for the PbS QD/c-DAE mixture. Two transient absorption bands at 488 and 560 nm emerge during the decay of the excited state of PbS QDs (Supplementary Fig. 4),

which is consistent with the observation of triplet c-DAE formation in our previous report.[21] Therefore, we conclude that TET process from QDs to c-DAE is the dominant mechanism responsible for both the sensitization of DAE photoisomerization and the reversible PL modulation process.

3. The instrumental details for the femtosecond transient absorption measurements have also been added to the Methods section on Page 20.

The fsTA measurement was carried out (TA-100, Time-Tech Spectra, Co., Ltd.) by using a Pharos2 fs-laser (Light Conversion; full width at half-maximum, ~222 fs, 100 kHz, 1030 nm), which was split into the pump and probe beams. The pump beam was directed into an optical parametric amplifier (OPA, Orpheus-HP, Light Conversion) to generate excitation pulses at 400 nm (1.2 nJ per pulse) or 780 nm (4 nJ per pulse), and was modulated at 500 Hz using an optical chopper for alternating pump-on and pump-off measurements. A broadband white-light continuum covering 380-600 nm (0.03 nJ per pulse) was generated by focusing a frequency-doubled 515 nm beam onto a sapphire plate. For the 500-900 nm probe range, the white-light continuum (0.05 nJ per pulse) was generated using a YAG crystal and filtered through a 900 nm low-pass filter. An optical delay stage (with maximum delay 7 ns) was used. The pump and probe beams were focused and overlapped on the sample in a 1 mm quartz cuvette. The transient spectra and kinetics were acquired by averaging 2,000 measurements at every given time delay.

2. *For real-world applications, the long-term stability of the solid-state samples under ambient conditions (oxygen, moisture) would be worth addressing. The current stability data (Fig. S8) is limited to 3 days in the dark. We suggest the authors perform the stability test for at least one week.*

**Reply to comment 1.2:**

We thank the Reviewer for emphasizing the importance of evaluating long-term stability of our samples under ambient conditions. Accordingly, the stability tests have been extended to seven days for green-emitting QDs combined with DAE and CdS QDs both in the solution and the solid-state. Furthermore, the NIR-emitting QDs combined with DAE and CdS QDs has also been examined over the same period in the solution. The results, provided as Supplementary Fig. 11-13, indicate that the samples retain photoswitchable and memory functions under ambient atmosphere in the dark at 24 °C and 25% relative humidity, highlighting their potential for practical applications.

**Our actions to comment 1.2:**

1. **Supplementary Fig. 11** has been modified to 7 days measurement as follow:

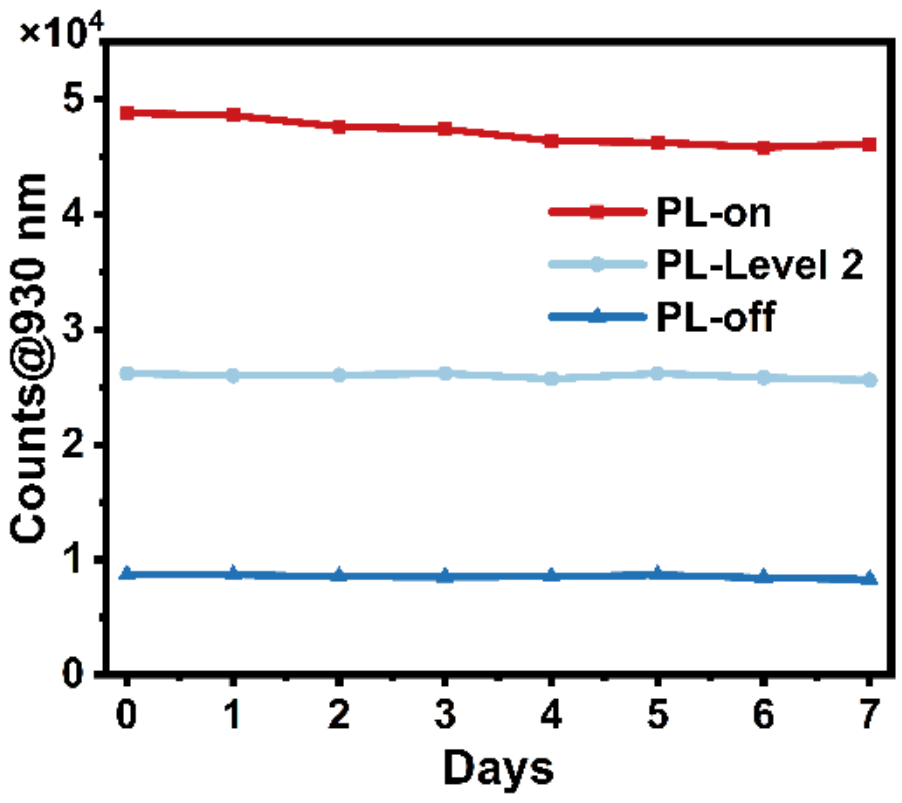


**Supplementary Fig. 11 Stability of three storage states for NIR emission samples.** PL intensity variation of on, level-2, and off states of the PbS QD/DAE/CdS QD sample over 7 days. All samples were stored in the dark under ambient atmosphere at 24 °C and 25% relative humidity.

2. **Supplementary Fig. 12** and **Supplementary Fig. 13** have been added as follow:

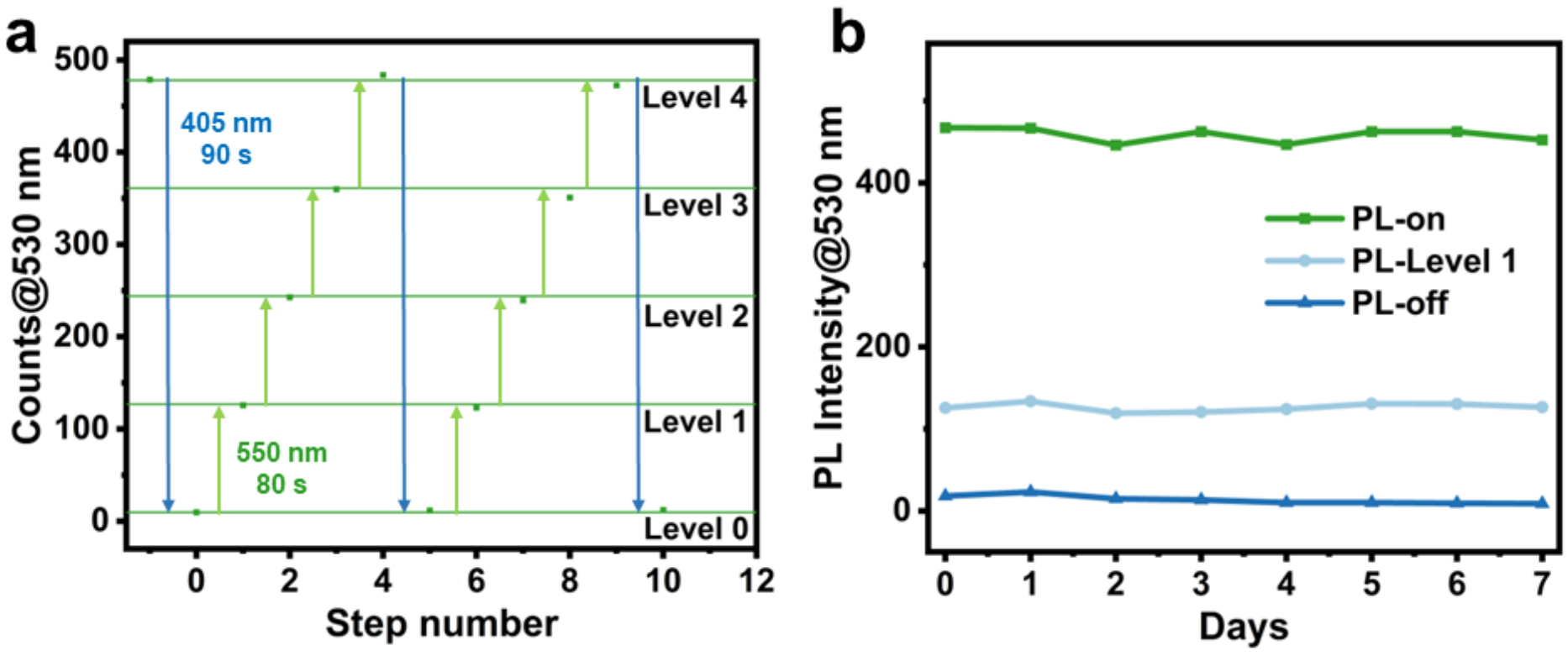


**Supplementary Fig. 12 Stability of different storage states in GQD/DAE/CdS QD solution samples. a** The signal at 530 nm was divided into 4 levels by an interval of 117 a.u. (defined as a single quantization level). The corresponding writing time for each level was set to 80 s under 550 nm light irradiation. **b** PL intensity variation of on, level-1, and off states of the GQD/DAE/CdS QD solution sample over 7 days.

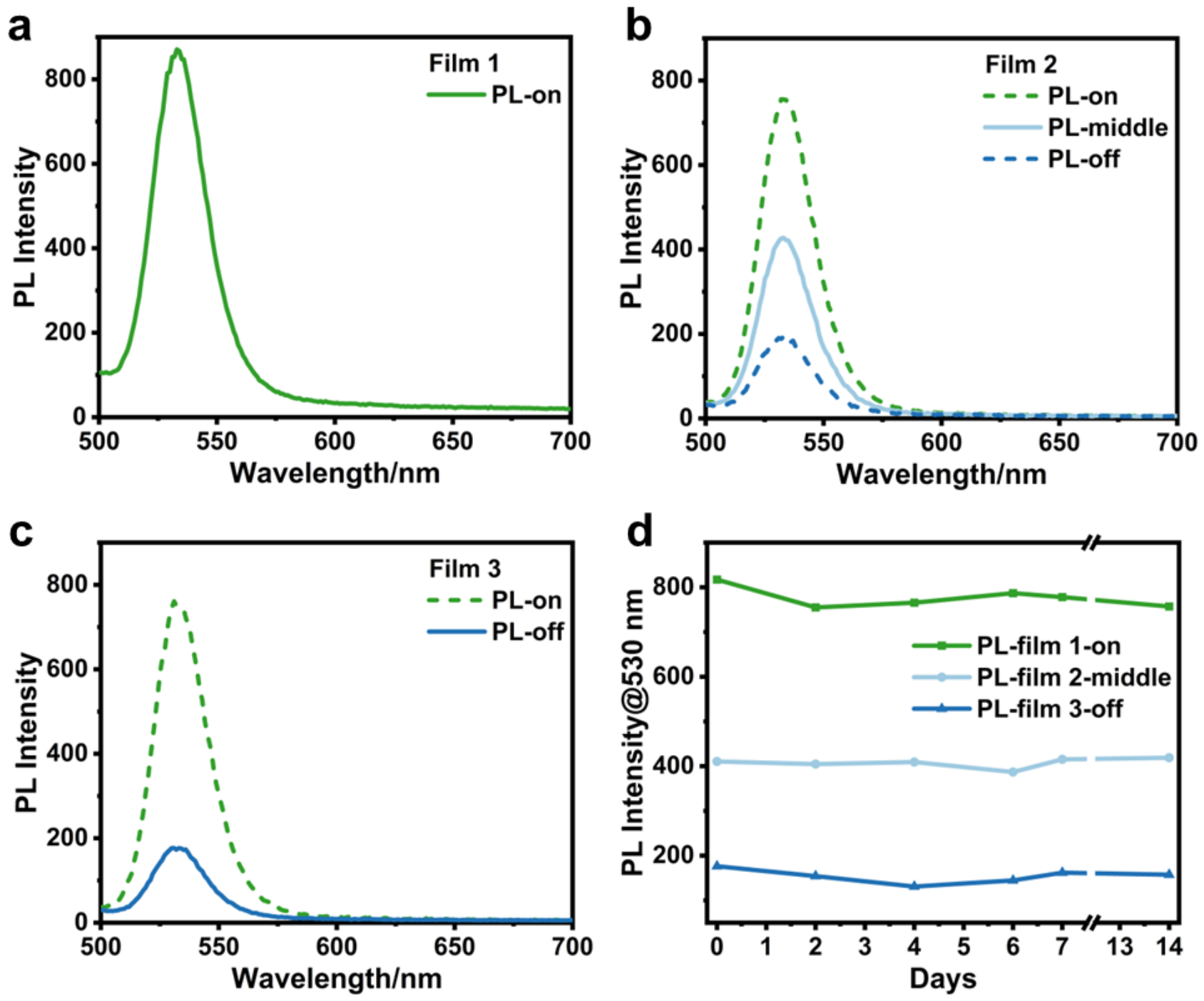


**Supplementary Fig. 13 Stability of different storage states in GQD/DAE/CdS QD thin films. a-c** PL spectra of three thin films representing the calibrated on, middle, and off states. **d** PL intensity variation of the corresponding storage states during storage for 14 days in the dark under ambient atmosphere at 24 °C and 25% relative humidity. The PL intensity of each state was referenced to the corresponding calibrated film.

3. On Page 13, now it reads as:

To evaluate the long-term stability of the photoswitching and memory functions towards practical applications, the PL intensities of PbS QD/DAE/CdS QD at three distinct memory states were monitored over 7 days under ambient atmosphere when stored in the dark at 24 °C and 25% relative humidity. As shown in Supplementary Fig. 11, the PL intensities of the on, level-2, and off states remain nearly constant throughout the storage period, demonstrating the excellent thermal stability of the memory states of our design under ambient conditions.

We also examined the memory functionality in the visible-emitting range, employing GQD/DAE/CdS QDs as a representative system for demonstration. The optical memory functionality was demonstrated using 550 nm light (104 mW/cm$^2$) for writing, 405 nm light for erasing, 480 nm excitation (0.38 mW/cm$^2$) for nondestructive reading, and the PL intensity of GQDs at 530 nm serving as the output signal. When an interval of 117 a.u. photon counts at 530 nm was defined as a single quantization level, a reversible green-emitting memory can be achieved with a data storage capacity of 4 levels (Supplementary Fig. 12a). Notably, GQD/DAE/CdS QD also exhibits excellent memory stability over 7 days storage (Supplementary Fig. 12b). Furthermore, after depositing GQD/DAE/CdS QD with polystyrene polymer onto an ITO substrate, the optical memory functionality was still retained in the solid state even after 14 days of storage (Supplementary Fig. 13), highlighting the potential for future optoelectronic data storage applications.

4. The experimental details for the fabrication of the data-storage films have also been added to the Methods section on Page 20.

Polystyrene was used as a film-forming polymer matrix. and mixed with GQD/DAE/PS/CdS QD solution (mass ratio = 1:0.2:1:5). The mixture was deposited onto an ITO substrate via dynamic spin coating at 2000 rpm for 40 s to form thin films for data storage. For the solid-state anti-counterfeiting sample, a mixed solution containing 400 µM CdS QDs, 2 mM o-DAE, and 20 µM PbS QDs was deposited onto a 30 mm × 30 mm piece of filter paper.

5. The details of 550 nm light source have been added to the Light Sources part in Methods section on Page 21.

3. *A table comparing this system's performance (spectral range, switching speeds, on/off ratios, fatigue) with previous UV-activated and visible-light-activated systems would help readers appreciate the advancements.*

**Reply to comment 1.3:**

We thank the Reviewer for this valuable suggestion. A comparative table has been added to the revised manuscript to enable the comparison between this work and previous UV light-activated systems. Key performance metrics are summarized in the table, including spectral range, switching speed, on/off ratio, and fatigue resistance.

**Our actions to comment 1.3:**

1. **Supplementary Table 3** has been added in the SI as follow:

**Supplementary Table 3**. Comparison of photoswitchable QDs combined with photochromic molecules reported in this work and previous studies.

| System | Spectral range (nm) | Switch-off* | Switch-on* | On/off ratio | Fatigue resistance | Ref |
|---|---|---|---|---|---|---|
| CdZnSeS gradient alloy QDs /DAE/ CdS QDs | 390-530 | 405 nm, 90 s, 19.1 mW/cm$^2$ | 515 nm, 3 min, 198 mW/cm$^2$ | 14.3 | 83.3% after 5 cycles | This work |
| | 500-600 | | | 250 | 90.2% after 5 cycles | |
| | 580-670 | | | 20.4 | ~100% after 5 cycles | |
| PbS QDs /DAE/ CdS QDs | 800-1100 | | | 15.6 | 69.3% after 5 cycles | |
| PbS QD/DAE | 700-900 | 302 nm, 60 s, 10 mW/cm$^2$ | 523 nm, 60 s, 15 mW/cm$^2$ | >100 | 4 cycles | 2022[3] |
| $CsPbBr_3$ QD/DAE | 480-550 | 365 nm, 6 min, 1.7 mW/cm$^2$ | 400-750 nm, 10 min, 13 mW/cm$^2$ | >100 | 3 cycles | 2022[4] |
| CdSe QD/ FFC | 525-675 | 365 nm, 27 min, 0.6 mW | 532 nm, -, 1.6 mW | ~5 | 6 cycles | 2021[5] |
| CdSe/ZnS QD/DAE | 500-600 | 300 nm, 10 s, 100 μW/cm$^2$ | 630 nm, 60 s, 150 mW/cm$^2$ | ~10 | - | 2020[6] |
| CdTe QD/Azo | 450-550 | 300 nm, - | Stored in dark, - | ~16 | 4 cycles | 2019[7] |
| $CsPbX_3$ QD/DAE | 420-500 | 365 nm, 5 min, 0.5 mW | >530 nm, 5 min, 150 mW | ~2 | 3 cycles | 2019[8] |
| | 480-560 | | | ~10 | | |
| | 530-700 | | | ~10 | | |
| CdSe/CdS/ZnS QD/DAE | 500-625 | 340 nm, 100 s, 1 mW/cm$^2$ | 550 nm, 40 s, 8.2 mW/cm$^2$ | ~1.67 | - | 2015[9] |
| CdSe/ZnS QD/DAE | 500-600 | 340 nm, 60 s, 1 mW/cm$^2$ | 545 nm, 60 s, 5.8 mW/cm$^2$ | ~1.5 | 16 cycles | 2011[10] |
| CdSe/ZnS QD/SP | 440-560 | 350 nm, 5 s, 0.97 mW | 588 nm, 90 s, 20 mW | ~8 | 4 cycles | 2005[11] |

* Switch off/on illumination parameters consist of incident light wavelength, irradiation time and optical power density.

2. On Page 12, it reads as:

The comparison with previously reported switchable QD systems under UV-light activation is summarized in Supplementary Table 3. The key performance parameters, such as PL wavelength range, activation light, PL on/off ratio, fatigue resistance, etc., are included in the table.

3. Corresponding references have been added in the SI:

3. Hou, L. *et al.* Optically switchable NIR photoluminescence of PbS semiconducting nanocrystals using diarylethene photoswitches. *J. Am. Chem. Soc.* **144**, 17758-17762 (2022).

4. Akaishi, Y. *et al.* Light-stimulated luminescence control of lead halide-based perovskite nanocrystals coupled with photochromic molecules via electron and energy transfer. *Small* **18**, 2205046 (2022).

5. Padgaonkar, S. *et al.* Light-triggered switching of quantum dot photoluminescence through excited-state electron transfer to surface-bound photochromic molecules. *Nano Lett.* **21**, 854-860 (2021).

6. Yano, N. *et al.* Photoluminescence on/off switching of a single colloidal quantum dot using photochromic diarylethene. *J. Phys. Chem. C* **124**, 17423-17429 (2020).

7. Saeed, S. *et al.* Photoresponsive azobenzene ligand as an efficient electron acceptor for luminous CdTe quantum dots. *J. Photoch. Photobio. A* **375**, 48-53 (2019).

8. Akaishi, Y. *et al.* Reversible ON/OFF switching of photoluminescence from CsPbX3 quantum dots coated with silica using photochromic diarylethene. *Chem. Commun.* **55**, 8060-8063 (2019).

9. Díaz, S.A. *et al.* Photoswitchable semiconductor nanocrystals with self-regulating photochromic Forster resonance energy transfer acceptors. *Nat. Commun.* **6**, 6036 (2015).

10. Díaz, S.A. *et al.* Photoswitchable water-soluble quantum dots: pcFRET based on amphiphilic photochromic polymer coating. *ACS Nano* **5**, 2795-2805 (2011).

11. Zhu, L.Y. *et al.* Light-controlled molecular switches modulate nanocrystal fluorescence. *J. Am. Chem. Soc.* **127**, 8968-8970 (2005).

4. *Some sections could benefit from tighter writing. For example, the introduction is somewhat lengthy and could be streamlined to focus more directly on the gap this work addresses.*

**Reply to comment 1.4:**

We thank for the Reviewer for this suggestion. The Introduction has been streamlined in the revised manuscript to improve conciseness and emphasize the research challenges addressed in this work.

**Our actions to comment 1.4:**

1. In the first paragraph on Page 3 of the main text, the sentence has been revised as:

Quantum dots (QDs), an ideal class of photoluminescence (PL) nanomaterials, possess broadband absorption,[8] narrow emission,[9] and size-tunable bandgap due to the strong quantum confinement effect.[10, 11] They have been widely applied in bioimaging,[12, 13] display,[14, 15] and

quantum photonic technology,[16, 17] and further coupling of PL on/off switching could further expand their potential in high-resolution imaging, optical memory, and encryption.

…

Therefore, combining QDs with molecular photoswitches provides a versatile strategy for constructing advanced light-responsive materials with dynamically tunable optical functionalities.

2. The discussion of recent research on QD-photoswitch systems on Page 3 has been streamlined as follow:

However, to date, all reported QD-photoswitch systems modulate only a single type of QD within a narrow spectral range due to the requirement of matched energy levels and/or spectral overlap. For instance, SP photoswitches and DAEs were employed to switch the green PL of CdSe/ZnS QDs through Förster resonance energy transfer (FRET) from QDs to the merocyanine or closed-form DAEs,[18, 19] CdSe QDs functionalized with furylfulgide photoswitches exhibited red PL on/off switch via photoinduced electron transfer process,[20] and DAEs anchored to PbS QDs enabled the near-infrared (NIR) PL switch through triplet energy transfer (TET).[21]

5. *Several latest researches on long-wavelength photoswitch design should be cited, e.g., JACS 2025, 5486; JACS, 2026, 6474.*

**Reply to comment 1.5:**

We thank the Reviewer for bringing these relevant and timely references to our attention. The suggested publications have been cited and discussed in the revised manuscript.

**Our actions to comment 1.5:**

1. On Page 4 in the main text, it reads:

Many research efforts have been contributed in developing all-visible-light activated molecular photoswitches via extending π-conjugation,[24] employing triplet sensitization,[25] or integrating upconversion nanoparticles[26]. For example, charge-transfer complex sensitizers were utilized to achieve bidirectional visible-light activated DAEs via triplet sensitization.[27] The rational design of perylene bisimide-based photoswitches enabled long-wavelength responsiveness with robust thermal bistability through triplet-mediated pathways.[28]

2. Corresponding reference provided by the Reviewer has been added:

27. Wang, W.H. *et al.* Vintages for new fashion: red-shifted photoswitching via the triplet-photoreaction channel with charge-transfer complex sensitizers. *J. Am. Chem. Soc.* **147**, 5486-5494 (2025).

28. Zhang, Z.W. *et al.* Thermo-bistable red and sensitized near-infrared photoswitches. *J. Am. Chem. Soc.* **148**, 6474-6484 (2026).

**Reviewer: 2**

*Comments:*
*This manuscript reports an interesting and efficient strategy that utilizes quantum dots (QDs) and photochromic molecular switches (Diarylethenes, DAEs) to reversibly switch photoluminescence on and off across a broad spectral range under visible light excitation. The authors also demonstrate their strategy for multi-level data storage and anti-counterfeiting in the NIR region, which holds promise for future smart light-responsive applications. Overall, this work represents a successful and excellent example of combining the fields of photoswitches and QDs, and should be of broad interest to researchers in relevant areas. The paper can be published in Nature Communications after addressing a few issues below:*

**Reply to general comment:**
We sincerely thank the Reviewer for the positive and encouraging assessment of our work, as well as for the thoughtful and constructive comments. In response, we have performed additional control experiments and QD triplet sensitization characterization, as well as further discussed future strategies in the revised manuscript and the SI. Our point-by-point responses to all comments are provided below:

1. *A control experiment containing only DAE and PbS QDs (or other QDs with strong photoluminescence) without CdS QDs should be performed. Importantly, the UV-visible absorption spectra should be monitored under purple light irradiation to clarify the role of CdS QDs as triplet sensitizers in achieving all-visible-light functions.*

**Reply to comment 2.1:**
The reviewer raises an important point on the role of CdS QDs as triplet sensitizers of DAEs. Accordingly, control experiments using only DAE and PbS QDs have been performed. The UV-vis absorption spectra were monitored under purple light irradiation (405 nm) and shown as Supplementary Fig. 6b in the revised SI. No significant spectral changes associated with DAE isomerization were observed in the absence of CdS QDs, which indicates that the photoswitching process cannot be triggered by the visible light without CdS QDs. This control experiment highlights the essential role of CdS QDs in enabling triplet energy transfer and all-visible-light activation. The corresponding results and discussion have been included in the revised manuscript.

**Our actions to comment 2.1:**
1. **Supplementary Fig. 6** has been added in SI as follow:

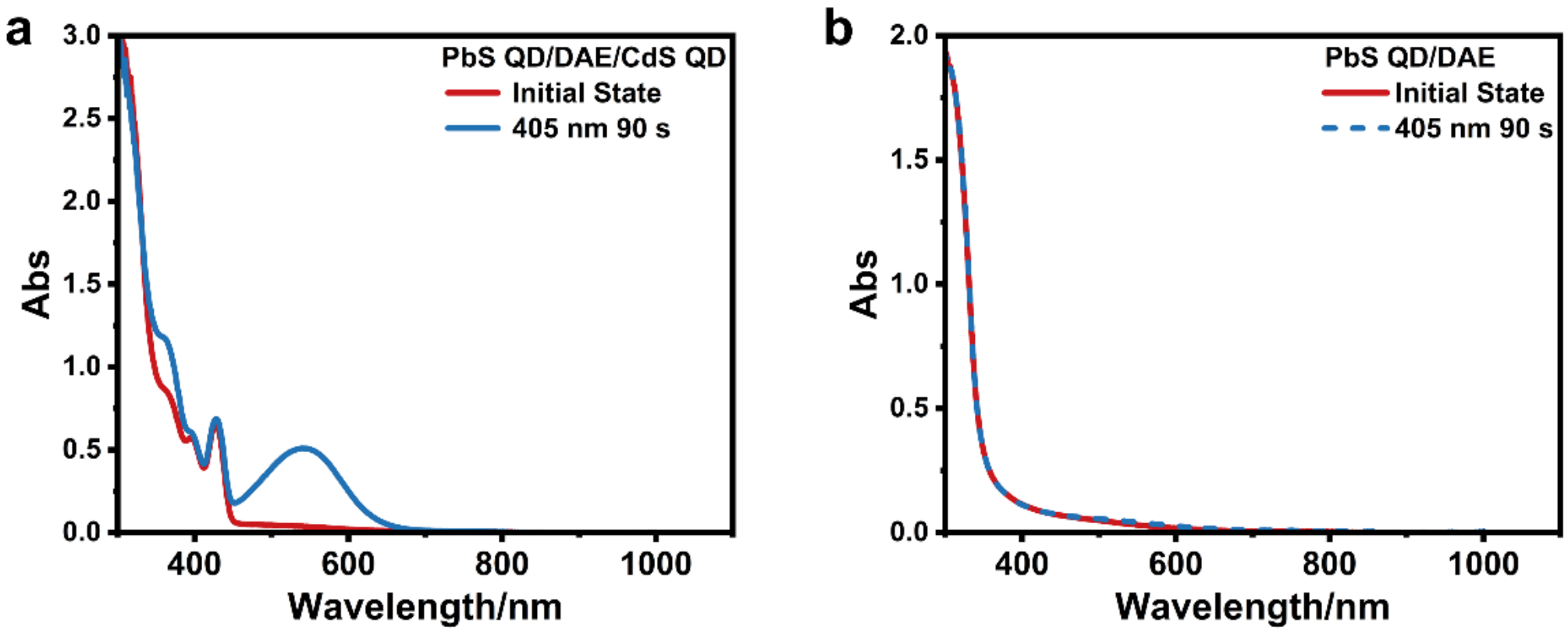


**Supplementary Fig. 6 UV-vis absorption spectra of QD/DAE mixture under 405 nm light irradiation.** UV-vis absorption spectra of the PbS QD/DAE mixture **a** with and **b** without CdS QDs under 405 nm irradiation for 90 s.

2. At the bottom of Page 9 in the main text, now it reads as:

In addition, in the absence of CdS QDs, the PbS QD/DAE mixture shows no spectral signatures of DAE isomerization under 405 nm light irradiation (Supplementary Fig. 6), indicating that CdS QDs act as essential triplet sensitizers enabling the ring-closing photoisomerization of DAEs under visible-light excitation.

2. *The x-scale titles appear to be cut off in Figure 2d-e, and error bars in Figure 2f should be provided and discussed.*

**Reply to comment 2.2:**

We are grateful for the Reviewer to point these issues out. The x-axis titles in Figures 2d-e have been corrected in the revised manuscript. In addition, error bars have been added to Figure 2f and 2i.

**Our actions to comment 2.2:**

1. **Figure 2** has been modified as follow:

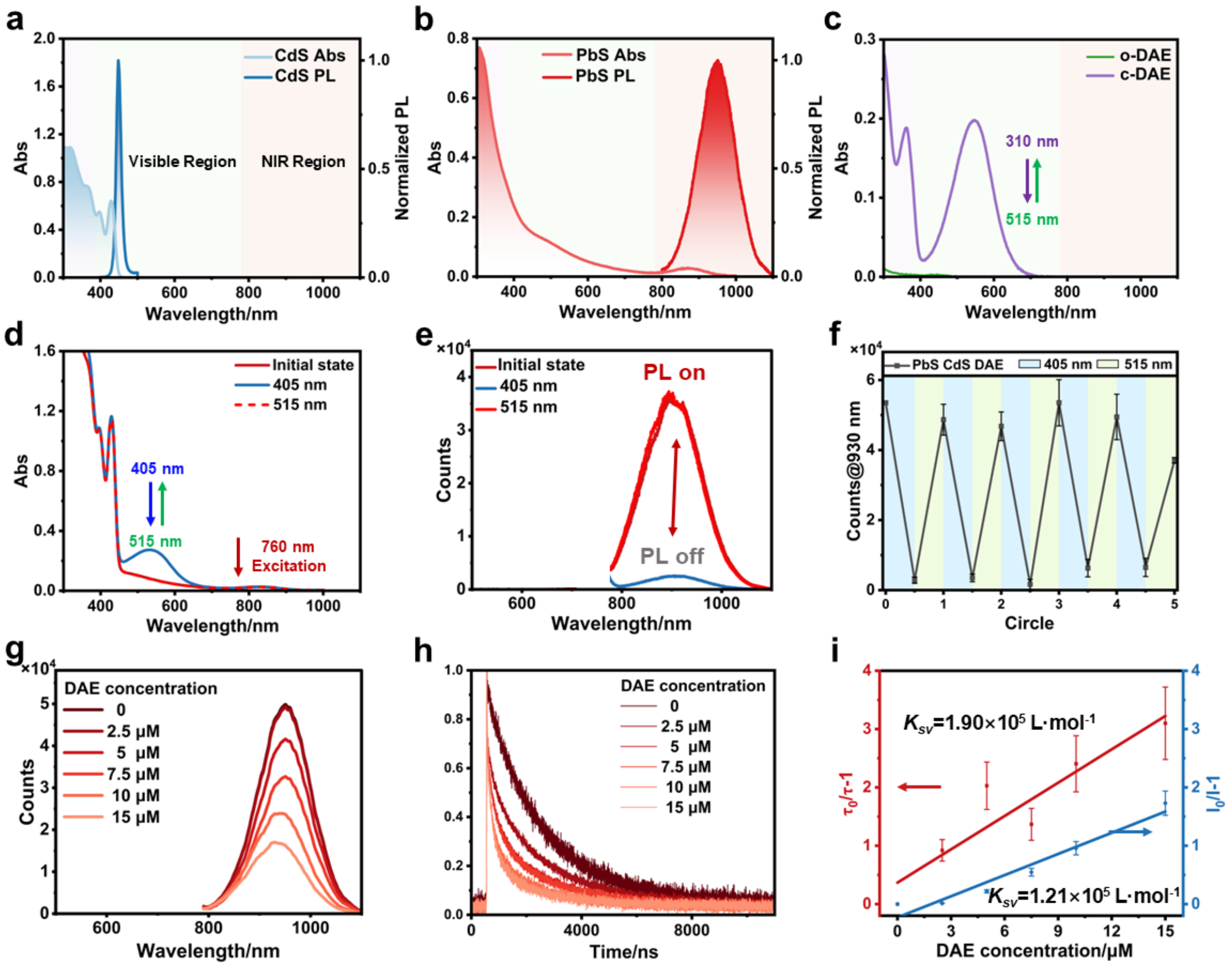


**Figure 2. Photophysical properties of the CdS QDs-DAE-PbS QDs system.** Absorption and PL spectra of **a** 5 µM CdS QDs and **b** 2 µM PbS QDs. **c** Absorption spectra of 100 µM DAE converted between o-DAE and c-DAE isomers upon UV and green light irradiation. **d** Absorption spectra and **e** PL spectra of the sample containing 8 µM CdS QDs, 160 µM DAE and 2 µM PbS QDs under 405 nm and 515 nm light irradiation. **f** Reversible PL modulation of PbS QDs demonstrated by the intensity at 930 nm over five cycles of alternating 405 nm and 515 nm light irradiation. **g** Steady-state PL spectra and **h** time-resolved PL decays of 2 µM PbS QDs with increasing c-DAE concentration (0, 2.5, 5, 7.5,10, and 15 µM). **i** Stern-Volmer plot of PL intensity quenching and lifetime quenching. All the samples were prepared in toluene, and the PL spectra in the NIR range has been corrected.

3. *Since triplet energy transfer (TET) is the key mechanism for achieving both visible activation of DAEs and photoswitching of QD photoluminescence in this work, relevant mechanistic studies in the field of QD triplet sensitization should be appropriately cited and discussed, such as J. Am. Chem. Soc. 2019, 141, 4186-4190; Nat. Commun. 2020, 11, 28; Nat. Mater. 2025, 24, 260-267.*

**Reply to comment 2.3:**

We thank the Reviewer for bringing these relevant studies on QD triplet sensitization to our attention. Accordingly, the recommended references have been incorporated and discussed, which provides a more comprehensive understanding of the mechanistic in this work and related field.

**Our actions to comment 2.3:**

1. On Page 9 in the main text, it reads:

Previous studies on QD sensitization of triplet molecules indicate that the efficient TET between QDs and organic molecules depends on donor-acceptor wavefunction overlap.[33, 34, 35] Therefore, it is essential to ensure close proximity between QDs and DAE molecules, which is achieved by introducing the carboxyl functional groups of DAEs to dynamically anchor to the surfaces of both types of QDs.

2. Corresponding reference provided by the Reviewer has been added:

33. Luo, X. *et al.* Triplet energy transfer from $CsPbBr_3$ nanocrystals enabled by quantum confinement. *J. Am. Chem. Soc.* **141**, 4186-4190 (2019).
34. Luo, X. *et al.* Mechanisms of triplet energy transfer across the inorganic nanocrystal/organic molecule interface. *Nat. Commun.* **11**, 28 (2020).
35. Liu, M. *et al.* Coherent manipulation of photochemical spin-triplet formation in quantum dot-molecule hybrids. *Nat. Mater.* **24**, 260-267 (2025).

4. *The authors should also discuss strategies to replace heavy metal-containing QDs to advance photoswitchable photoluminescence toward biocompatible applications.*

**Reply to comment 2.4:**

We agree with the Reviewer that the development of more environmentally friendly systems is an important future direction of our design. In response, we have added a discussion in the conclusion part of the revised manuscript on potential strategies to replace heavy metal-containing QDs, highlighting emerging heavy-metal-free QDs as promising alternatives.

**Our actions to comment 2.4:**

On Page 17, the sentence has added as follow:

Furthermore, extending this strategy to heavy-metal-free QDs, such as ZnSe QD, InP QD or $CuInSe_2$ QD-based systems, may broaden the scope of applications.

# Point-by-point Reply

Dear Reviewers,

Thank you for taking time to carefully review our manuscript. We are grateful for your recognition and positive assessment of our revised manuscript.

**Reviewer: 1**

*Comments:*
*The authors have fully addressed the issues raised by all reviewers. The manuscript is much improved in current form and we are happy to read the revised version and to see the efforts the authors made. We consider the present version could meet the quality to be accepted by Nature Communications. Thank you!*

**Reply to general comment:**
We sincerely thank the Reviewer for the encouraging comment and the recognition of our efforts. We are grateful for the Reviewer's careful assessment and support of the revised work.

**Reviewer: 2**

*Comments:*
*The authors have carefully conducted additional experiments (including transient spectroscopy, stability and control measurements) to address reviewers' comments. The revised manuscript is suitable for publication.*

**Reply to general comment:**
We sincerely thank the Reviewer for recognizing the additional experiments conducted to address the comments. We greatly appreciate the Reviewer's careful evaluation and support of our work.
